# A Metamaterial Computational Multi‐Sensor of Grip‐Strength Properties with Point‐of‐Care Human‐Computer Interaction

**DOI:** 10.1002/advs.202304091

**Published:** 2023-10-11

**Authors:** Yinghua Chen, Tianrun Li, Zhemin Wang, Zhimiao Yan, Raffaella De Vita, Ting Tan

**Affiliations:** ^1^ State Key Laboratory of Mechanical System and Vibration School of Mechanical Engineering Shanghai Jiao Tong University Shanghai 200240 P. R. China; ^2^ State Key Laboratory of Ocean Engineering Department of Mechanics School of Naval Architecture Ocean & Civil Engineering Shanghai Jiao Tong University Shanghai 200240 P. R. China; ^3^ Department of Biomedical Engineering and Mechanics Virginia Tech Blacksburg VA 24061 USA

**Keywords:** grip strength, human‐computer interaction, mechanical metamaterials, piezoelectricity, self‐powered sensing

## Abstract

Grip strength is a biomarker of frailty and an evaluation indicator of brain health, cardiovascular morbidity, and psychological health. Yet, the development of a reliable, interactive, and point‐of‐care device for comprehensive multi‐sensing of hand grip status is challenging. Here, a relation between soft buckling metamaterial deformations and built piezoelectric voltage signals is uncovered to achieve multiple sensing of maximal grip force, grip speed, grip impulse, and endurance indicators. A metamaterial computational sensor design is established by hyperelastic model that governs the mechanical characterization, machine learning models for computational sensing, and graphical user interface to provide visual cues. A exemplify grip measurement for left and right hands of seven elderly campus workers is conducted. By taking indicators of grip status as input parameters, human‐computer interactive games are incorporated into the computational sensor to improve the user compliance with measurement protocols. Two elderly female schizophrenic patients are participated in the real‐time interactive point‐of‐care grip assessment and training for potentially sarcopenia screening. The attractive features of this advanced intelligent metamaterial computational sensing system are crucial to establish a point‐of‐care biomechanical platform and advancing the human‐computer interactive healthcare, ultimately contributing to a global health ecosystem.

## Graphical abstract

A reliable, interactive, and point‐of‐care device sensing maximal grip force, grip speed, grip impulse, and endurance indicators for multi‐sensing comprehensive grip strength status is developed. Implemented with interactive games with tactile and visual feedback to increase user interest and compliance, the sensor advances arising fields of point‐of‐care computational sensing, human‐machine interactive system and internet of medical things.

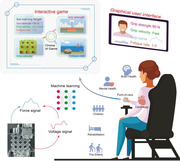

## 1 Introduction

Grip strength is a crucial biomarker due to its relevance to frailty,^[^ ^1^ ^]^ aging,^[^ ^2^ ^]^ cardiovascular morbidity and mortality,^[^ ^3^ ^]^ the degree of recovery from heart surgery,^[^ ^4^, ^5^, ^6^ ^]^ brain health,^[^ ^7^, ^8^ ^]^ and mental health.^[^ ^9^ ^]^ In addition to maximum grip strength, other important indicators of overall health are grip speed, grip impulse, and fatigue rate derived from the grip force‐time curve. These grip parameters complement each other to provide a more comprehensive assessment of the grip strength status. Finger dexterity related to grip velocity^[^ ^10^, ^11^, ^12^ ^]^ is linked to various medical conditions such as Alzheimer's disease,^[^ ^13^ ^]^ Parkinson's disease,^[^ ^14^ ^]^ schizophrenia,^[^ ^15^ ^]^ stroke,^[^ ^16^ ^]^ cervical myelopathy (CM),^[^ ^17^ ^]^ as well as post‐operative recovery after single finger replantation,^[^ ^18^ ^]^ repair of flexor tendons in the finger zone‐II,^[^ ^19^ ^]^ and the developmental status of adolescents.^[^ ^20^ ^]^ Specifically, individuals with Alzheimer's disease exhibit significantly lower finger agility compared to healthy elderly individuals.^[^ ^13^ ^]^ In the progression of Parkinson's disease, issues with finger agility become more pronounced.^[^ ^14^ ^]^ Impairment of motor agility itself is considered a hallmark of chronic schizophrenia, independent of cognitive function.^[^ ^15^ ^]^ After a stroke, grip abilities of the fingers are impaired relative to normal conditions.^[^ ^16^ ^]^ Analysis of gripping motion effectively and objectively assesses the current impairment of hand agility in CM patients, aiding in determining disease severity, progression, and the efficacy of surgical intervention.^[^ ^17^ ^]^ Furthermore, finger agility plays a role in evaluating post‐operative motor skills after finger replantation surgery,^[^ ^18^ ^]^ assessing the recovery of finger zone‐II flexor tendon functionality,^[^ ^19^ ^]^ and offering insights for developmental intervention and treatment in adolescents.^[^ ^20^ ^]^ Grip strength endurance, indicated by grip strength pulses and fatigue rates, can serve as a predictive indicator for conditions such as chronic obstructive pulmonary disease (COPD),^[^ ^21^ ^]^ fibromyalgia syndrome (FM),^[^ ^22^ ^]^ rheumatoid hands,^[^ ^23^ ^]^ inflammation in hospitalized geriatric patients,^[^ ^24^ ^]^ and multiple sclerosis.^[^ ^25^ ^]^ It can be assessed using multiple metrics, such as the area under the curve (AUC),^[^ ^21^, ^22^, ^23^ ^]^ fatigue rate (FR = area above the curve / area below the curve),^[^ ^26^ ^]^ time taken for grip strength to decline to 50%,^[^ ^24^, ^27^, ^28^ ^]^ 60%,^[^ ^29^ ^]^ or 70%^[^ ^30^ ^]^ of maximum grip strength, and static fatigue index.^[^ ^25^ ^]^ Specifically, AUC in COPD patients decreased by 28% compared to the control group (*P* = 0.001), demonstrating a superior prognostic value compared to the 6‐minute walk test in COPD prognosis.^[^ ^21^ ^]^ AUC in female FM patients was notably lower than in healthy females (*p* < 0.001).^[^ ^22^ ^]^ Rheumatoid hand deformities, such as swan neck and combined deformities, lead to reduced AUC due to weakened grip strength.^[^ ^23^ ^]^ Elderly patients with systemic inflammation upon hospitalization exhibited greater weakness and diminished anti‐fatigue capacity compared to non‐inflammatory patients.^[^ ^24^ ^]^ In patients with multiple sclerosis, the static fatigue index was significantly higher than in the healthy control group.^[^ ^25^ ^]^Presently, commercially available spring‐loaded and hydraulic grip measurement devices are primarily capable of recording maximum grip strength and are often devoid of data storage capabilities. In contrast, grip measurement devices, whether commercially available or not, based on sensor technology, when integrated with computers, have the capacity to capture real‐time grip strength values and incorporate data storage functionalities. Nevertheless, the majority of current commercial or non‐commercial grip measurement devices predominantly assess isometric grip strength, thereby offering limited ability to directly reflect grip velocity.^[^ ^31^, ^32^, ^33^, ^34^, ^35^, ^36^ ^]^ Therefore, multiple sensing for maximum grip force, grip speed, and grip impulse is needed for a comprehensive assessment of grip strength.

Biomedical measurement devices that incorporate human‐computer interactive games that feature tactile and visual feedback of the target acquisition increase user interest and incentive as well as user compliance. Carrying out interactive grip strength measurements regularly can serve as a medical rehabilitation training method for preventing and delaying onset of cardiovascular, neurodegenerative, and psychological diseases.^[^ ^4^, ^8^, ^9^ ^]^ For instance, the middle‐aged and older adults who switch from working to non‐working status are at a higher risk of reduced grip strength than others and require muscle strength training interventions to improve grip strength and prevent sarcopenia.^[^ ^37^ ^]^ Future neurorehabilitation therapies also focus on the competence to precisely control the decline in grip strength and the improvement of movement quality to some extent.^[^ ^38^ ^]^ Exergames inherently involve cognitive training and even simple exergames without explicit cognitive demands essentially require cognitive processing.^[^ ^39^, ^40^, ^41^ ^]^ Grip measurements assisted by interactive games have the potential to improve cognitive performance in patients with neurodegenerative diseases.

**Figure 1:**
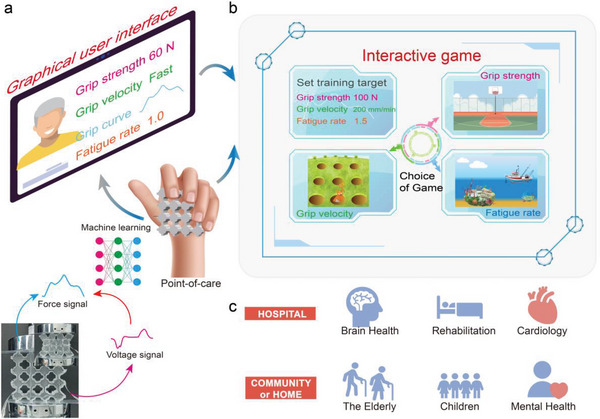
Design principle and application scenarios of the metamaterial piezoelectric computational biosensing system for grip‐strength. a) Schematic of the graphical user interface and schematic of the computational sensing. b) Schematic representation of the interactive game interface used to train different grip strength metrics. c) Biomedical targeted population and venue for grip‐strength point‐of‐care treatment.

Monitoring and improving the hand grip properties in healthy people and patients require the development of a reliable, lightweight, maintenance‐free, interactive, and multi‐sensing device. In this study, a buckling‐based soft metamaterial is designed as the structural matrix material to introduce a reversible and predictable pattern transformation.^[^ ^42^, ^43^, ^44^, ^45^, ^46^ ^]^ Piezoelectric films of polyvinylidene fluoride (PVDF) are deformed in coordination with the metamaterial to generate stable and distinctive voltage signals in response to grip force stimuli.^[^ ^47^, ^48^, ^49^, ^50^ ^]^ Machine learning (ML) algorithms are introduced to capture the corresponding relationship between the voltage signals generated by the piezoelectric material and the mechanical characteristics of the loaded structural metamaterial (**Figure** **1**a).^[^ ^51^, ^52^, ^53^, ^54^ ^]^ The resulting computational grip multi‐sensor provides a reliable, self‐powered, maintenance‐free measurement of grip status, encompassing the evaluation of maximum grip strength to gauge muscle strength, assessment of gripping speed to reflect finger agility, and analysis of grip fatigue indicators such as the area under the force‐time curve and fatigue rate to portray grip endurance. Grip characteristics such as maximum grip strength, grip speed, and fatigue rate derived from the grip force‐time curve can be measured and altered using video games with adjustable targets (Figure 1b). Monitoring grip strength and postoperative recovery in patients with cardiovascular and neurological diseases in hospitals, as well as supervising grip strength in patients with mental illness in psychiatric centers and in vulnerable populations like children and the elderly in communities or at home can be made feasible due to the mobility and portability of the proposed multi‐sensing device (Figure 1c). The biometric monitoring device for grip strength can serve as a point‐of‐care tool in rehabilitation training, thus contributing to a global health ecosystem.

## 2 Results

### 2.1 Design and Characterization

The buckling‐based mechanical metamaterial is created by tiling tessellation of the fundamental cell with the four‐leaf‐clover‐like void at the intersection (MM‐F, see Figure S1a, Supporting Information). For electro‐mechanical comparison, the metamaterial with the circular voids of the same porosity as the four‐leaf‐clover‐like one is used as the control group (MM‐C). A mathematical description of the void design for MM‐F and MM‐C is given in the Note S1 (Supporting Information). The mechanical metamaterials used for grip‐strength assessment are designed with a palm‐fit dimension of 8 × 8 × 1.8 cm^3^ and fabricated by casting polydimethylsiloxane (PDMS) in the 3D printed molds and demolding after curing (Figure S1b, Supporting Information, and Figure 1c, see the Experimental Section for a detailed description of the fabrication).

**Figure 2:**
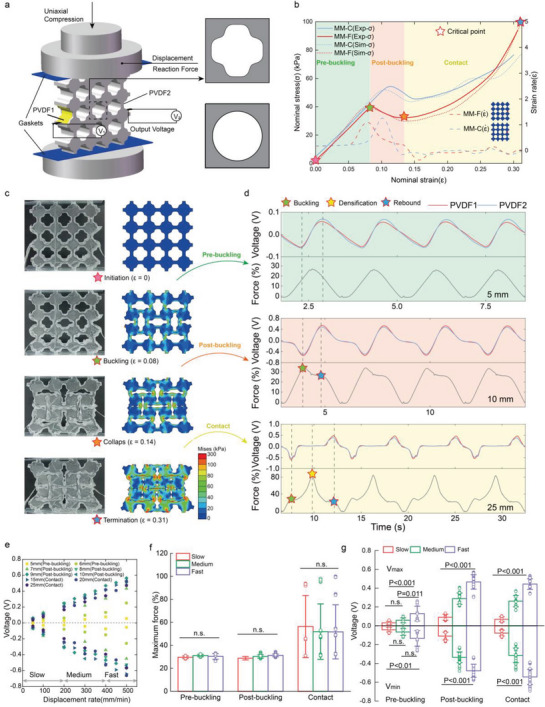
Mechano‐electric characterization of the MM‐F. a) Experimental apparatus for quasi‐static uniaxial compression tests. b) Experimental and simulated nominal stress‐strain curves of the MM‐F and MM‐C specimens at the constant deformation rate of 500 mm min^−1^, and simulated strain rate at the midpoint on the right side of the two metamaterials varied by the nominal strain. c) Experimental and simulated snapshots of the MM‐F at the initiation, buckling, collapse and the end. d) Time histories of the voltage and force and final experimental snapshots with the compression displacements of 5, 10, and 25 mm at the displacement rate of 500 mm min^−1^ under cyclic loading and unloading. e) The values of the maximum and minimum voltages for PVDF1 under different compression displacements and compression velocities. f) The maximum forces and g) the minimum and maximum voltages for PVDF1 of four cycles under different types of compression speed and state. Mean values are shown and error bars represent ± s.d. (*n*=16–40 samples per group), as analyzed by one‐way ANOVA with post hoc *t*‐tests with Bonferroni correction.

In order to leverage the nonlinear mechanical response of the metamaterial for computational sensing, fundamental mechanical and electro‐mechanical properties are characterized. The mechanical characterization of the metamaterial was experimentally conducted by the quasi‐static uniaxial compression tests (**Figure** **2**a). The MM‐F and MM‐C specimens demonstrate similar nominal stress‐strain behaviors consisting of three typical regions (solid lines in Figure 2b): an initial linear elastic region (pre‐buckling), followed by a stress‐decreasing region (post‐buckling) and a final densified hardening region (contact). The simulated nominal stress‐strain curves of the two metamaterials using the finite element method with the Yeoh hyperelastic model (dot lines in Figure 2b) are consistent with the experimental results. An imperfection in the form of the first buckling mode determined in advance by linear buckling analysis with a scale factor of 1% was introduced to prevent the simulation from following an unstable deformation path (see the Experimental Section for a detailed description of the simulation).^[^ ^55^ ^]^ The initial linear elastic responses of the two metamaterials are almost identical because of the same matrix material and porosity. The disappearance of the linear elastic response is the consequence of buckling, indicated by the stress peak before the stress valley. The simulated strain rates at the side‐midpoints of the metamaterials (dash lines in Figure 2b), where the PVDF films are to be integrated with (see the Methods), reach their maximum values at each corresponding buckling point. This is consistent with the experimental observations that buckling occurs instantaneously compared to pre‐buckling and post‐buckling deformations (Video S1, Supporting Information). Owing to the dramatic variation in the strain rate, it is reasonable to anticipate a high voltage output by the buckling of the piezoelectric material. Once the buckling occurs, the stress‐decreasing region with negative stiffness appears and the unit cells of the mechanical metamaterial display rotational behaviors (Figure 2c). More experimental and simulated snapshots are presented in Figure S2 (Supporting Information). The simulation results show that the Mises stress is primarily distributed in ligaments between cells, particularly at the midpoints of those, under the uniaxial compression with the negative Poisson's ratio of −1. Subsequently, the internal pores collapse, and their boundaries begin to contact under high strain, causing the final steep region of the nominal stress‐strain curve.

To harvest the mechanical energy of the metamaterial compression and generate the electrical signals by piezoelectricity, two PVDF films were integrated respectively on the ligaments covering the midpoints of the left and right sides of the MM‐F and MM‐C, where the peaks of the stress and strain rate occur with buckling. Results show that the MM‐F generates the same level of voltage as the MM‐C under a smaller force and exhibits higher output consistency (Note S2, Supporting Information, experimental reaction force and open‐circuit voltage results of the MM‐F and MM‐C specimens can be seen in Figure S3a, Supporting Information). The consistency originates from the compact capability of the MM‐F to maintain a high level of symmetry under large deformations,^[^ ^56^, ^57^ ^]^ which is not possessed by the MM‐C. Consequently, the MM‐F is selected to be the matrix metamaterial in this study. During 200 cycles of loading and unloading, the MM‐F demonstrates consistent and durable electro‐mechanical characteristics (Figure S4a,b, Supporting Information). The voltage outputs of the PVDF1 and PVDF2 are in phase, allowing connection in series to produce a multiplicative voltage (Figure S4c, Supporting Information).

Cyclic loading and unloading experiments were carried out under different experimental conditions to explore the effects of displacement and displacement rate on the electro‐mechanical characterization. See for Note S4 (Supporting Information) for detailed experimental parameters. According to the buckling and collapse points previously observed in the mechano‐electric characterization of the MM‐F, we classified the compression state into three types, namely, pre‐buckling (between initiation and buckling), post‐buckling (between buckling and collapse), and contact (between collapse and termination). Figure 2d presents force and voltage curves of the three compression states at the same displacement rate of 500 mm min^−1^. Forces have been normalized using *F*_max_ = 26.26 N, the maximum force measured in all the experiments, for more convenient comparison of mechanical responses under different experimental conditions. For the pre‐buckling state, the voltage amplitude is small, and the force‐time curve has one decreasing stage initiated by unloading. For the post‐buckling state, the voltage‐time curve has a clear plateau transition between the maximum voltage in the previous period and the minimum voltage in the subsequent period, and the force‐time curve displays two stages of force decreasing initiated by the buckling and rebound. For the contact state, the voltage‐time curve displays a long platform between the negative and positive peaks, and the force‐time curve displays three stages of force decreasing initiated by the buckling, densification, and rebound. See Figure S5 (Supporting Information) for the measured force and voltage varied with the time under other compression displacements.

For pre‐buckling state, the absolute values of the maximum and minimum voltages both grow with the increase of compression displacement, which corresponds to the escalating trend of the strain rate varied by the strain before buckling. While for post‐buckling and contact states, the maximum and minimum voltages resulting respectively from buckling and elastic recovery almost do not vary with the displacement (Figure 2e). To assess the impact of grip speed which can be used to judge the state of recovery at finger joints and grip motion control ability of the basal ganglia,^[^ ^38^, ^39^, ^58^ ^]^ the displacement rate of the metamaterial piezoelectric prototype is classified into three grades: slow (50–200 mm min^−1^), medium (200–400 mm min^−1^), and fast (400–500 mm min^−1^) (Figure 2e; Figure S6a, Supporting Information). The maximum force of the same compression state under different levels of speed differs rarely (Figure 2f). However, with the increase of the displacement rate, the absolute values of the maximum and minimum voltages of PVDF1 (Figure 2g) and PVDF2 (Figure S6b, Supporting Information) of the same compression state experience significant increases. For pre‐buckling state, the voltage difference between adjacent speeds is not very significant (n.s. or *P* = 0.011), but the difference between fast and slow speeds is significant (*P* <0.01 or *P* <0.001). For post‐buckling and contact states, the differences between pairs for all three speeds are significant (*P*<0.001).

### 2.2 Computational Multi‐Sensing

**Figure 3:**
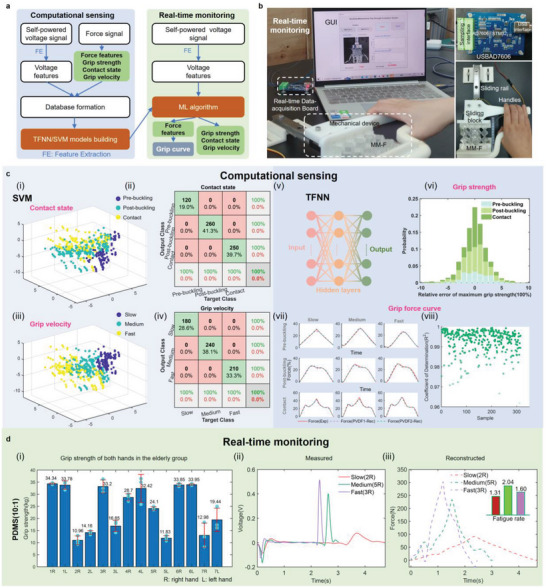
Machine learning assisted force sensing and real‐time monitoring. a) Flow chart of computational sensing and real‐time monitoring. b) The experimental apparatus for real‐time monitoring. c) The PCA clustered results for (i) contact state and (iii) grip velocity. The corresponding confusion map for (ii) contact state and (iv) grip velocity. The recognition accuracies of training set (90%) and test set (10%) are both 100%. (v) Schematic diagram of TFNN. (vi) Probability distribution of relative errors in maximum grip strength measurements. (vii) Force curves reconstructed from voltage data of PVDF1 and PVDF2 and curves obtained experimentally under three types of contact state and three types of compression speed. (viii) Coefficient of determination *R* ^2^ between the original force curve and the reconstructed force curve for all samples of PVDF2. d) (i) Grip strength of both hands for seven elderly participants using MM‐F (PDMS 10:1). Measured voltage signal (ii), reconstructed grip strength curve (iii), and derived fatigue rate for right hands of participants 2, 5, and 3. 2R = right hand of the participant 2.

The flowcharts of computational multi‐sensing and real‐time monitoring of the hand grip properties are presented in **Figure** **3**a. The constructed mechanical metamaterial piezoelectric device, composed of a metamaterial and piezoelectric material PVDF integrated on both sides of the metamaterial, is self‐powered in that it can generate voltage signals for actively sensing the grip stimuli applied to it (See Note S5 and Figure S7, Supporting Information, about the self‐powered mechanism). The above analyses illustrate the nonlinear relationships between the grip information and the voltage signals generated by piezoelectricity. Machine learning (ML) has recently been introduced to calibrate sensors with nonlinear characteristics for data sensing.^[^ ^60^ ^]^ The multilayer feedforward neural network is suitable and sufficient for multiple regression tasks and thus it is used to map the piezoelectric signal features to the continuous responses of the grip force and relative characteristics;^[^ ^61^, ^62^, ^63^ ^]^ the support vector machine (SVM) exhibits excellent performance in classification tasks and is utilized to distinguish the deformation states and speed levels; principal component analysis (PCA) processing is the most commonly used unsupervised linear dimensionality reduction method (Note S5, Supporting Information).^[^ ^64^, ^65^, ^66^ ^]^ In real‐time monitoring, voltage signals of the prototype piezoelectric metamaterial with computational sensing capability are obtained by a real‐time data‐acquisition board (Figure 3b). Multi‐type grip information of the grip strength, velocity grade, and fatigue rate (impulse) with real‐time interface display is attainable after the signals are processed by the machine learning algorithm.

To verify that the deformation states and speed levels can be distinguished for the metamaterial piezoelectric sensor, the preliminary classification results using PCA for data reduction with obvious aggregation are visualized in Figure 3c(i),(ii). A similar result could be achieved when we evaluate the input voltage characteristics utilizing multi‐class SVM classifier through the one‐against‐one split principle and the Gaussian kernel for further identification. In addition, Error Correcting Output Codes (ECCO) technology is applied to enable the classifier to have certain fault tolerance. It can be seen from the confusion matrix that the recognition accuracies for the three types of deformation and the three speed levels are both 100% (Figure 3c(iii),(iv)).

Based on the discrepant characteristics of the three categories for force‐voltage relations, a two‐layer feedforward neural network (TFNN) with 21 sigmoid hidden neurons and linear output neurons is employed to fit the maximum grip force and further obtain the relevant data required to reconstruct the grip force‐time curve (Figure 3c(v)). The training results demonstrate that the relative error between the output maximum force and the actual target maximum force is 0.01 ± 2.23% (mean ± std) and ranges between −3.61% and 3.68% (95th percentiles) (Figure 3c(vi)). The linear correlation coefficient between the target force and output force is greater than 0.99 (Figure S8a, Supporting Information), and the absolute error is −0.01 ± 0.23 N (mean ± std) and ranges between −0.36 N and 0.33 N (95^th^ percentiles) (Figure S8b, Supporting Information).

To reconstruct the grip force‐time curve, the output of the TFNN should be tuned to be 100 data points in time series for the force data. However, it is difficult and time‐consuming to fit such high‐dimensional data leveraging TFNN. After dimensionality reduction, the 100 data points in time series for force data are reduced to 15 with a cumulative contribution rate of more than 99.9% for information, indicating that they can depict well the original waveform. A mapping from the open‐circuit voltage to the compressive force with high dependence on the displacement rate and state is constructed. The flow chart for reconstructing the force‐time curve is shown in Figure S9 (Supporting Information). Training results show that the linear correlation coefficient between the output (15 main features of force) of the neural network after training and the actual target output is 0.99 (Figure S10a, Supporting Information). Through the 15 output features from the neural network, we can refactor the force waveform. The force waveform for each electromechanical classification reconstructed by this method for PVDF1 and PVDF2 differs rarely from the original force waveform (Figure 3c(vii)). The coefficients of determination R^2^ between the original force curve and the reconstructed force curve using the voltages of PVDF2 are greater than 0.96 (Figure 3c(viii)), indicating an excellent reconstruction performance. See Figure S10b (Supporting Information) for the *R* ^2^ using the voltages of PVDF1. See Figure S11 (Supporting Information) for 63 sets of the reconstructed force‐time curves. The above results show that the metamaterial piezoelectric computational multi‐sensor has the potential to precisely reconstruct the grip curve that can be used to extract more useful biomedical indicators.

Since the relationship between output and input was calibrated using the standard force data set of the static machine and the standard voltage data set of data acquisition device, the reliability of the machine learning models to the real application scenarios can be examined according to whether the deformation state inferred corresponds to the actual deformation state. Via the above trained machine learning algorithm, the deformation state, grip speed, maximum grip force, and reconstructed grip force curve can be displayed in a graphical user interface (GUI, Note S7, Supporting Information) on the monitor after the subject grasps the handle of the device during real‐time grip strength monitoring (see the Experimental Section for detailed real‐time data acquisition).

An exemplify hand grip status measurement is conducted on seven elderly people working on campus. A mechanical metamaterial (PDMS 10:1) multi‐sensing device is fabricated (Mechanical response can be seen in Figure S12, Supporting Information) and calibrated using the above ML methods (details are presented in Note S7, Supporting Information). The fair performance of ML models can be seen in Figure S14 (Supporting Information). Grip‐strength‐related factors involving gender, height, weight, and health status of the participants are provided in the Table S1 (Supporting Information).^[^ ^67^, ^68^, ^69^, ^70^ ^]^ Four participants are female and three are male, and three participants are left‐handers. The age of the participants is 58 ± 5 years old, the body weight is 62.85 ± 5.79 kg, and the body height is 163.14 ± 5.05 cm. All the contact states sensed by our device are consistent with those actually observed. Grip strengths at maximum performance (Figure 3d(i)), fatigue rates (FR, defined as the ratio of the area above the curve to the area below the curve, Figure S15, Supporting Information), areas under the grip curve (AUC, Figure S16, Supporting Information), grip velocities (Figure S17, Supporting Information) and other indicators of grip endurance (Figure S18–S21, Supporting Information) based on three measurements of the two hands are obtained. Among the cohort of seven elderly participants, four female subjects (57 ± 3.56 years old) exhibited a grip strength (the maximum value from both hands) of 27.54 ± 6.93 kg, while three male subjects (60 ± 6.25 years old) demonstrated a grip strength of 27.48 ± 11.54 kg, closely correspond with outcomes reported in prior global studies on grip strength.^[^ ^71^ ^]^ Results show a significant influence of body weight (Pearson *r* = 0.88, *p* = 0.009) on the grip strength of the dominant hand (27.36 ± 8.15 kg) (Figure S22, Supporting Information), while grip strength endurance of the dominant hand, assessed through fatigue rate, exhibited no correlation with maximum grip strength (Pearson *r* = 0.03, *p* = 0.956), consistent with previous research findings.^[^ ^28^, ^72^, ^73^ ^]^ Except for participants 2 and 5 whose partial grip velocities are slow which may be related to cholecystectomy or hypertension, all participants exhibit a moderate or fast grip speed. The grip velocity of all three measurements for right hand of participants numbered 2, 5, and 3 is slow, medium, and fast, respectively. The corresponding measured voltage and reconstructed grip strength curves within our expectations during the first measurement are shown in Figure 3d(ii),(iii). See Figures S23 and S24 (Supporting Information) for GUIs of the above three categories of test results and those under other compression patterns and speeds. We observed that participants 2, 5, and 7 consistently exhibited slow or medium grip velocities, which could suggest compromised finger flexibility and the need for grip strength training to prevent conditions such as Alzheimer's disease, Parkinson's disease, and chronic schizophrenia.^[^ ^13^, ^14^, ^15^ ^]^ Furthermore, we analyzed various indicators reflecting grip endurance. According to the area under the curve (AUC) metric, we found that the non‐dominant hand AUC of participants 3, 4, and 7 was lower compared to other participants, indicating a higher risk of chronic obstructive pulmonary disease (COPD) and rheumatoid hand disorders.^[^ ^21^, ^23^ ^]^ Particularly, these three participants were female, indicating an increased risk of fibromyalgia syndrome (FM).^[^ ^22^ ^]^ When examining the time that takes for grip strength to decline to 50%, 60%, or 70% of maximum grip strength as an indicator of grip endurance, we observed that participants 2 and 5 generally exhibited longer times, potentially indicating a risk of inflammation.^[^ ^24^ ^]^ The fatigue rate and static fatigue index among the 7 participants showed minimal differences, suggesting similar risks of multiple sclerosis.^[^ ^25^ ^]^

The measuring range of the sensor can be customized by adjusting the Young's modulus of the mechanical metamaterial, e.g., changing the ratio of the main agent to the curing agent of PDMS. For instance, the metamaterial piezoelectric computational sensor made by PDMS of 30:1 displays excellent performance in measuring grip strength under 2.68 kg, which is suitable for weak individuals such as those with a grip strength of less than 1 kg after stroke or surgery and sick children.^[^ ^34^, ^35^ ^]^

### 2.3 Human‐Computer Interactive Testing

**Figure 4:**
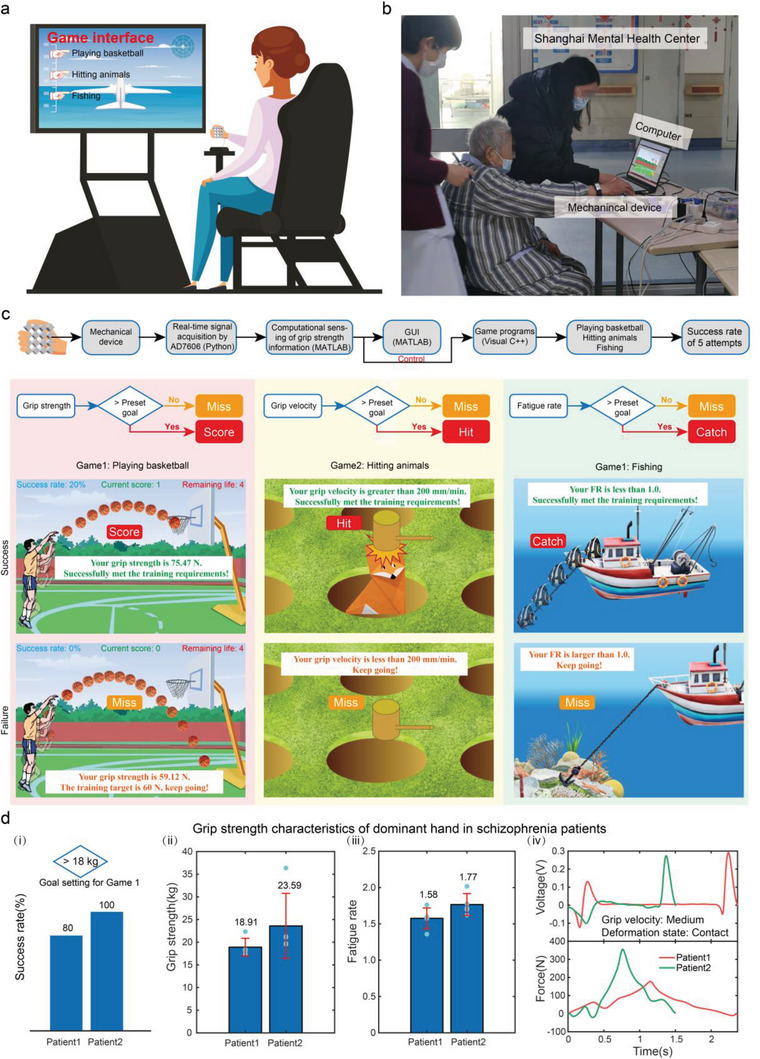
Interactive games for rehabilitation training. a) Scenario diagram of grip strength characteristics monitoring based on real‐time interactive games. b) Picture of grip strength characteristics measurement for schizophrenia patients in Shanghai Mental Health Center accompanied by a medical worker. c) Flow chart of the technological path from the point of care device to the implementation of multi‐sensing of hand grasp with human‐computer interaction. Logical flow diagrams and screenshots of successful and failed interfaces for three interactive games consisting of *Playing Basketball*, *Hitting Animals*, and *Fishing*. d) Success rate (i), grip strength (ii), fatigue rate (iii) for five measurements, and measured voltage and reconstructed force (iv) during the first measurement for two patients.

Common people have sufficient cognitive ability and initiative to perform grip strength measurements. However, patients with schizophrenic diseases perform worse on cognitive and daily functional skills than common counterparts.^[^ ^74^ ^]^ Therefore, monitoring grip strength characteristics is combined with well‐designed interactive games to improve compliance of subjects with poor cognitive ability, as shown in **Figure** **4**a,b. When the subject grasps the handles, the grip information obtained from real‐time data acquisition can be displayed on the GUI or used as input to control game programs (Figure 4c). Based on the Visual C++ platform, we build three grip interactive games: *Playing Basketball*, *Hitting Animals*, and *Fishing* to measure or train the subjects' grip strength, grip speed, and fatigue rate, respectively (Video S3, Supporting Information). The effectiveness of training is measured through the success rate of five attempts for a certain game. *Playing Basketball* requires a controlled grip strength. If the subject's grip strength exceeds the preset goal, the avatar in the game will make a shot, otherwise the shot will be missed (Figure 4c). As we would expect, success becomes increasingly difficult as the threshold increases. *Hitting Animals* focus on controlled grip velocity. If the subject's grip speed exceeds the preset threshold, the randomly presented critter will be hit, otherwise it will escape (Figure 4c). *Fishing* uses fatigue rate to control a hook and a line to reel a fish in. If the subject's fatigue rate is less than the setting goal, the fish will be dragged onto the boat, otherwise the fish will break loose (Figure 4c).

Sarcopenia is more prevalent in people with schizophrenia than in the general population.^[^ ^75^ ^]^ The diagnosis of sarcopenia relies on measurements of muscle strength and muscle mass. Grip strength is often used to assess muscle strength. The European Working Group on Sarcopenia in Older People recommends that the cut‐off point for low grip strength be 16 kg for female and 27 kg for male.^[^ ^76^ ^]^ The threshold recommended by the Asian Working Group for Sarcopenia is 18 kg for female and 28 kg for male.^[^ ^77^ ^]^ Grip strength characteristics of two female schizophrenic patients in Shanghai Mental Health Center were tested with the setting goal for grip strength in *Playing Basketball* being 18 kg. The success rate is shown in Figure 4d(i). The results show that patient 1 is at risk for sarcopenia. The grip strength and fatigue rate of the two patients for five measurements are also recorded and stored, as shown in Figure 4d(ii),(iii). The corresponding measured voltage and reconstructed grip strength curves for the first measurement are shown in Figure 4d(iv). Based on the recorded grip strength curves, we have also obtainedthe grip velocity, the area under the curve, the static fatigue rate, the amount of time it takes for grip strength to drop to 50%, 60%, or 70% of maximum grip strength for two female schizophrenic patients (Figure S25, Supporting Information). Regarding the 2 patients with schizophrenia, their dominant hand's grip velocity consistently remained at a medium level, possibly due to compromised motor flexibility associated with schizophrenia.^[^ ^15^ ^]^ As for grip endurance, patient 1 demonstrated longer times to reach 50%, 60%, and 70% of maximum grip strength compared to patient 2. Additionally, patient 1 exhibited a lower area under the curve and static fatigue index than patient 2, suggesting superior grip endurance. Regular human‐computer interactive grip strength measurement can serve to not only monitor grip strength, but to also carry out grip strength rehabilitation training. By presetting the training goals, we can achieve customized grip training for individuals with different grip strength characteristics. The training device provides interactive rehabilitation training that is supported by real‐time data that are convenient to remote transmission. This opens new possibilities for the establishment of point‐of‐care treatment and Healthcare Internet‐of‐Things.^[^ ^78^ ^]^

## 3 Conclusion

This work demonstrates the development of a real‐time interactive metamaterial computational sensing device that can monitor multiple grip properties and improve user compliance. Through long‐term regular monitoring of grip properties via this device, new muscle strength rehabilitation training protocols can be designed. Specifically, the device can measure different people's hand grip characteristics such as the maximum grip force, the grip strength curve, and the grip velocity. Additional work needs to be done to turn the ideas presented here into a commercialized medical device. The tunability of mechanical properties for the metamaterial^[^ ^42^ ^]^ facilitates the versatility of the device which can account for the palm size and grip strength range of different subjects. Given the effectiveness of rehabilitation training, new devices that can help in personalized rehabilitation training plans for patients are helpful and can help follow the recommendations of professional doctors. Continuous storage of human health data like grip strength can also be analyzed by experts through machine learning techniques for disease prevention, diagnosis, and intervention.^[^ ^79^ ^]^ The hand grip measurement results can be combined with gait,^[^ ^80^ ^]^ eye movement,^[^ ^40^ ^]^ bioelectrical impedance,^[^ ^81^ ^]^ and other data for clinical modeling, early warning and diagnosis platform of mental diseases, nervous diseases, and Sarcopenia. Furthermore, in the future, piezoelectric energy harvesting devices and wireless transmission modules can be integrated into our designed device to enhance its practical application value.^[^ ^82^, ^83^, ^84^, ^85^ ^]^ Overall, these attractive features of the intelligent mechanical metamaterial device presented here have the potential to impact the biomedical field and healthcare delivery, advancing wireless point‐of‐care platforms and the internet of medical things.

## 4 Experimental Section

### Specimen Fabrication

Mechanical metamaterials were fabricated by casting PDMS (Sylgard 184, Dow Corning Co., Ltd) into a 12‐part mold. The mold parts designed in SOLIDWORKS are 3D‐printed with white resin (R4600, WeNext Technology Co., Ltd.). The main agent and curing agent of PDMS were mixed with a mass ratio of 30:1 and stirred for 15 min before being put into a vacuum chamber for defoaming treatment. To demonstrate the possibility of measuring a wider range of grip strength, the agent mass ratio was adjusted to 10:1. The liquid degassed PDMS material was then poured into the mold. Tapes were used to seal the four sides and bottom of the mold to prevent the liquid from leaking through small gaps. The 184‐release agent (Wuxi Fites Electronic Technology Co., Ltd) was also applied to the mold during the casting stage for easier removal. Because the casting process was exposed to the atmosphere, it was difficult to avoid the re‐formation of bubbles, hence after casting, the whole mold needed to be degassed with a pump and a vacuum chamber again before it was moved into the dryer to cure. Given that the thermal deformation temperature of the white resin is 44–57 °C, the drying temperature was set to 40 °C. After curing for 48 h, the main body of the metamaterial geometry was obtained by demolding. The PVDF films (Shen Zhen Vkinging Electronics Co., Ltd) are rectangle elements of the piezoelectric film with silver ink screen printed electrodes. Rather than making the lead attachment near the sensor, the piezo polymer tail extended from the active sensor area as flex circuit material with offset traces, which gave a very flat, flexible lead with a connector at the end. In addition, the films had a tape release layer adhesive in the sensor area. The thickness of the piezoelectric film was 28 µm and the polarization direction was the thickness direction. The piezoelectric strain constants *d_31_* and *d_33_* were 23 × 10^−12^ C N^−1^ and −33 × 10^−12^ C N^−1^, respectively. The PVDF films were bonded to the middle‐side concave of the mechanical metamaterial for generating voltage signals under force stimuli.

### Mechanical Experimental Characterization

The specimens were placed on a material machine (HY‐0580, Shanghai Hengyi Precision Instrument Co., LTD) for the quasi‐static experiment, and two acrylic plates with thickness of 2 mm were placed above and below the specimens. The material testing machine had two load cells with capacity of 10 N and 500 N, respectively, and effective force measurement resolution of 0.1% and displacement and speed measurement accuracy of 0.5% (Transcell Technology, Inc.). With a constant displacement rate from a load frame, the compression displacement *u* and the force *F* on specimens were measured by the machine. The uniaxial nominal strain is calculated from *ε = u/L* where *L* is the macroscopic original length of the specimen. The uniaxial nominal stress was calculated from *σ = F/A* where *A* is the macroscopic original cross‐section area of the specimen. For MM‐F, *A* = 263.52 mm^2^. For MM‐C, *A* = 326.16 mm^2^. Two PVDF films were connected to two channels of the data acquisition device (DH5922D, Donghua Testing Technology Co., Ltd.) respectively to collect the voltage signals of PVDF1 and PVDF2. The sampling frequency of all displacement and force data used in experiments was 50 Hz, and that of voltage data was 500 Hz.

### Finite Element Modeling

ABAQUS 2020 (Dassault Systems Simulia.) Linear‐Perturbation Buckling and Dynamic‐Explicit simulations were conducted with 2D planar and deformable models based on the constant material cross‐section. Yeoh hyperelastic material model was used to capture the mechanical response of a PDMS‐fabricated cubic with a side length of 2 mm under uniaxial compression. The strain energy density for Yeoh model^[^ ^86^ ^]^ can be expressed as $U\;=\sum_{i=1}^{3}C_{i0}{\left(I_{1}-3\right)}^{i}\;+\sum_{i\;=\;0}^{3}1/D_{i}\;{\left(J-1\right)}^{2i}$, where *C_i0_* (*C_10_* = 22.75 kPa, *C_20_* = 0.15 kPa, and *C_30_* = 0.09 kPa) is the material constant obtained experimentally and *D_i_* (*D_i_* = 0) is the material constant related to the compressibility of the material, *I_1_* is the first‐order strain invariant and *J* is the volume ratio, which both can be expressed in terms of the deformation gradient **F as** *I_1_* = trace(**F F** ^T^) and *J* = det(**F**). The 6‐node modified, quadratic, and plane‐strain triangular elements (CPE6M) were used to mesh the specimens. For the Linear‐Perturbation Bucking, the Lanczos Eigen solver was used to solve the first buckling mode and the bottom boundaries of the specimen were fixed and a small compressive displacement was applied at the top boundary of the specimen. For the Dynamic‐Explicit simulation, a period of 2.4 s that was proportional to the quasi‐static experimental loading rate was used. The upper and lower gaskets were also modeled. A linear elastic model with Young's modulus of 210 GPa and Poisson's ratio of 0.3 was used for the gaskets that were not shown in the simulation diagram in the body. The 4‐node bilinear, plane‐stress quadrilateral, and reduced‐integration elements (CPS4R) were used to mesh the gaskets. The constraint of a rigid body was applied to both the upper and lower gaskets. The bottom boundary of the lower gasket was fixed, and a compressive displacement of 20 mm was applied to the top boundary of the upper gasket. A general contact with a tangential friction coefficient of 0.1 was used. A surface‐to‐surface contact with a tangential friction coefficient of 0.5 was used between the specimen and two gaskets.

### Real‐Time Data Acquisition Method

The data‐acquisition board (USB2AD7606, Shenzhen Anyibo Electronic Technology Co., LTD) had eight‐channel data sampling interfaces and a USB interface, and its maximum sampling frequency was 200 kHz. One of data sampling interfaces was connected to a piece of PVDF film. The sampled data obtained for secondary analysis was transferred to a monitor through the USB interface. To facilitate subjects' grip and ensure that the mechanical apparatus does not affect the metamaterial's performance, we designed the handle gripper, as illustrated in Figure 3b. The handle gripper consists of fixed and movable fixtures. On the side opposite to each other, both the fixed and movable fixtures possess horizontal support platforms. The mechanical metamaterial piezoelectric prototype is placed parallelly on the horizontal support platforms of the movable and fixed fixtures. The fixed fixture is affixed to an optical platform, while the movable fixture is mounted on the slider of the guide rail. One end of the guide rail is attached to the fixed fixture, and the other end is secured to the optical platform using support components. The guide rail ensures unidirectional linear motion of the movable fixture, maintaining consistency with the unidirectional linear motion during static mechanical testing. Both the fixed and movable fixtures establish surface contact with the upper and lower surfaces of the metamaterial, respectively. Moreover, two fixtures' contact surfaces are composed of a rigid material (resin), resembling the contact conditions with the metamaterial during static mechanical testing. A comparison of the experimental conditions between actual testing and static mechanical testing is outlined in Table S2 (Supporting Information). During data acquisition, the metamaterial piezoelectric prototype was placed parallel to the horizontal support platform of the moving fixture and the fixed fixture whose handles were designed to match the posture of the human fingers when grasped. When the subject grasped the handles, the grip force was transferred to the mechanical metamaterial via the linear constraints of the slider guide. The PVDF film integrated in the metamaterial converted the force signals to electrical signals. The packaged AD7606 data acquisition module then transmitted the electrical data to the serial port of the monitor through USB serial communication, which could realize high‐speed data transmission. Programs in the Python environment were compiled to call the DLL library functions encapsulated in the data acquisition module (a collection of fully encapsulated functions in the Windows environment) to achieve data reading and storage.

### Establishment of a Graphical User Interface

The experiment only measured the grip strength of the subjects and did not cause any harm to their health. Subjects took part with informed consent. MATLAB R2021b (MathWorks, Inc.) was used to build the GUI that displayed information concerning subjects' grip information (See Note S6, Supporting Information). During the measurement of the grip strength information, the subject had 10 seconds to grasp the handles. Simultaneously, MATLAB called the Python code for data collection, and loaded the trained machine learning models to process the data, and displayed the grip information on the GUI.

### Rehabilitation Training Process

Rehabilitation training game programs were written in visual C++ in advance. When rehabilitation training was carried out, the subjects grasped the handles within 10 s according to the prompt. At the same time, visual C++ called MATLAB code for data acquisition and data processing. If the subject achieved the training goal, the related animation of game success was shown. After 5 times of attempt, the success rate of the relevant game was displayed.

### Statistics and Reproducibility

For MM‐F (PDMS 30:1), by virtue of facilitating data processing for training and testing, 63 sets of raw voltage and force data previously obtained were broken up into 630 samples through time normalization and segment. Consequently, a sample contained a single cycle of data from a single PVDF film, which comprised of 1000 data points in time series for the voltage data and 100 data points in time series for the force data. Twenty‐one characteristic indexes in time domain and frequency for the voltage data, including curve features (e.g., maximum and minimum values corresponding to information about grip strength, grip speed, deformation state, etc.) were extracted to substitute 1000 potentially redundant data points. The full list of 21 characteristic indexes in time domain and frequency for voltage data^[^ ^87^ ^]^ included maximum value, minimum value, mean value, peak‐to‐peak value, corrected mean value, variance, standard deviation, kurtosis, skewness, root mean square factor, waveform factor, peak factor, pulse factor, margin factor, the center of gravity frequency, mean square frequency, root mean square frequency, frequency variance, frequency standard deviation, power spectrum entropy, and singular spectrum entropy.

### Statistical Analysis

Results were expressed as the mean ± SD as specified. Mean differences were analyzed by one‐way ANOVA with post hoc *t*‐tests with Bonferroni correction. A value of *p* < 0.05 was regarded as statistically significant. The presence of a linear correlation between two variables was assessed using Pearson correlation analysis. value of *p* < 0.05 was considered indicative of a statistically significant correlation. Data were analyzed with OriginPro 2023b software.

## Conflict of Interest

The authors declare no conflict of interest.

## Supporting information

Supporting Information

Supplemental Video 1

Supplemental Video 2

Supplemental Video 3

## Data Availability

The data that support the findings of this study are available from the corresponding author upon reasonable request.
