# Supplementary material for: A Metamaterial Computational Multi‐Sensor of Grip‐Strength Properties with Point‐of‐Care Human‐Computer Interaction
Source: Adv Sci (Weinh). 2023 Oct 11;10(34):2304091. doi: 10.1002/advs.202304091 (PMC10700692; doi:10.1002/advs.202304091)
Supplement: Supplementary file 1 — Supporting Information [file ADVS-10-2304091-s001.pdf]

ADVANCED SCIENCE
Open Access

Supporting Information

for *Adv. Sci.*, DOI 10.1002/advs.202304091

A Metamaterial Computational Multi-Sensor of Grip-Strength Properties with Point-of-Care Human-Computer Interaction

*Yinghua Chen, Tianrun Li, Zhemin Wang, Zhimiao Yan, Raffaella De Vita and Ting Tan**

Supporting Information

A Metamaterial Computational Multi-sensor of Grip-strength Properties with Point-of-care Human-computer Interaction

*Yinghua Chen[1], Tianrun Li[1], Zhemin Wang[1], Zhimiao Yan[2], Raffaella De Vita[3], Ting Tan[1]**

Supplementary Note 1: Mathematical description of voids for MM-F and MM-C

The representative volume elements (RVEs) for the two metamaterials (MM-F and MM-C) considered in this work are shown in Figure S1a. The outline of the voids for the two metamaterials can be expressed by Fourier series expansion:

$$x = r_\theta \cos\theta\,,\ y = r_\theta \sin\theta$$

$$\text{with } r_\theta = r_0[1 + c_1 cos(4\theta) + c_2 cos(8\theta)]$$

where θ ranges from 0 to 2π, and three parameters, c_1， c_2 and r_0 control the shape of the pore (61,62). For MM-C, as shown by the dotted line, there are

$$c_1 = c_2 = 0$$

For MM-F, there are

$$c_1 = 0.11，\ c_2 = -0.05$$

The side length of the metamaterial is designed to be 80mm to fit the size of a human palm and 4×4 cells are sufficient to reflect the mechanical behavior of the metamaterial and facilitate the integration of the piezoelectric films. Therefore, the square length L_0 take 20 mm. The porosity ϕ_0 for this work is 0.5. And r_0 can be determined by the porosity ϕ_0:

$$r_0 = \frac{L_0\sqrt{2\phi_0}}{\sqrt{\pi(2 + c_1^2 + c_2^2)}}$$

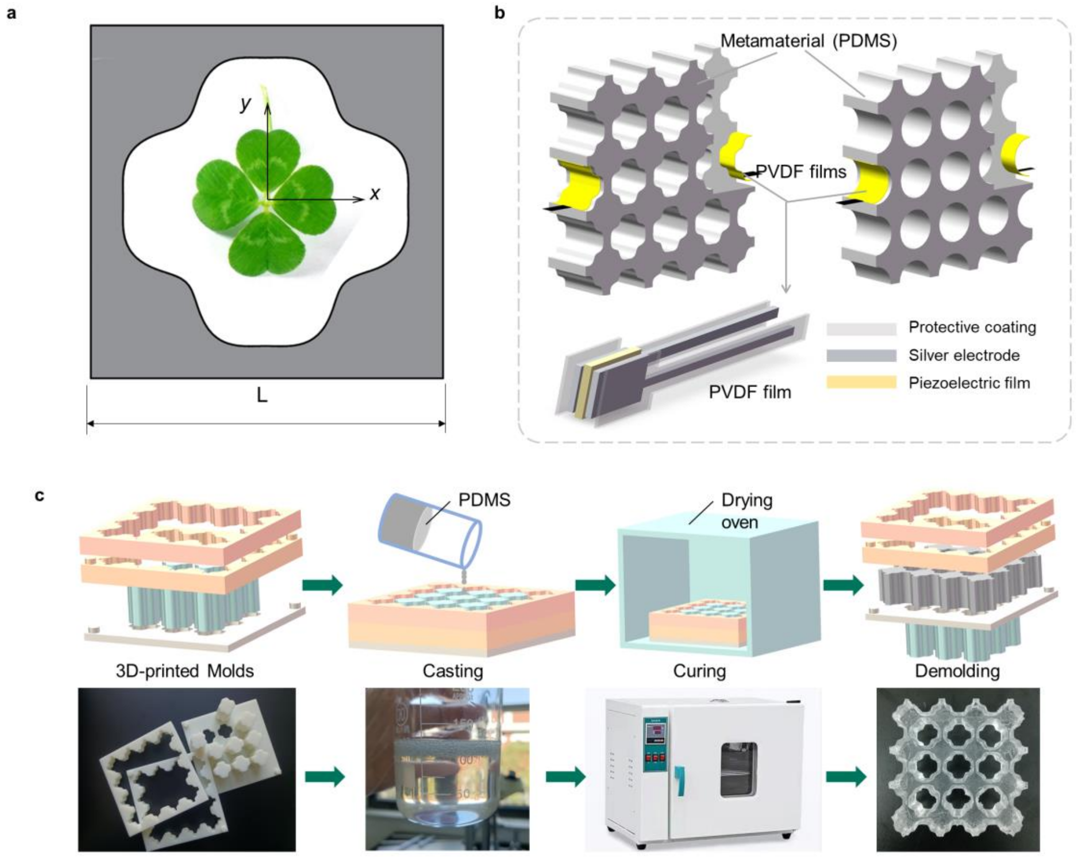

Figure S1. a Representative volume element (RVEs) for the two metamaterials considered in this work. The solid line is MM-F voids, the dotted line is MM-C voids. **b** Schematic diagram of MM-F and MM-C samples. **c** Four-step mold casting procedure for the specimens.

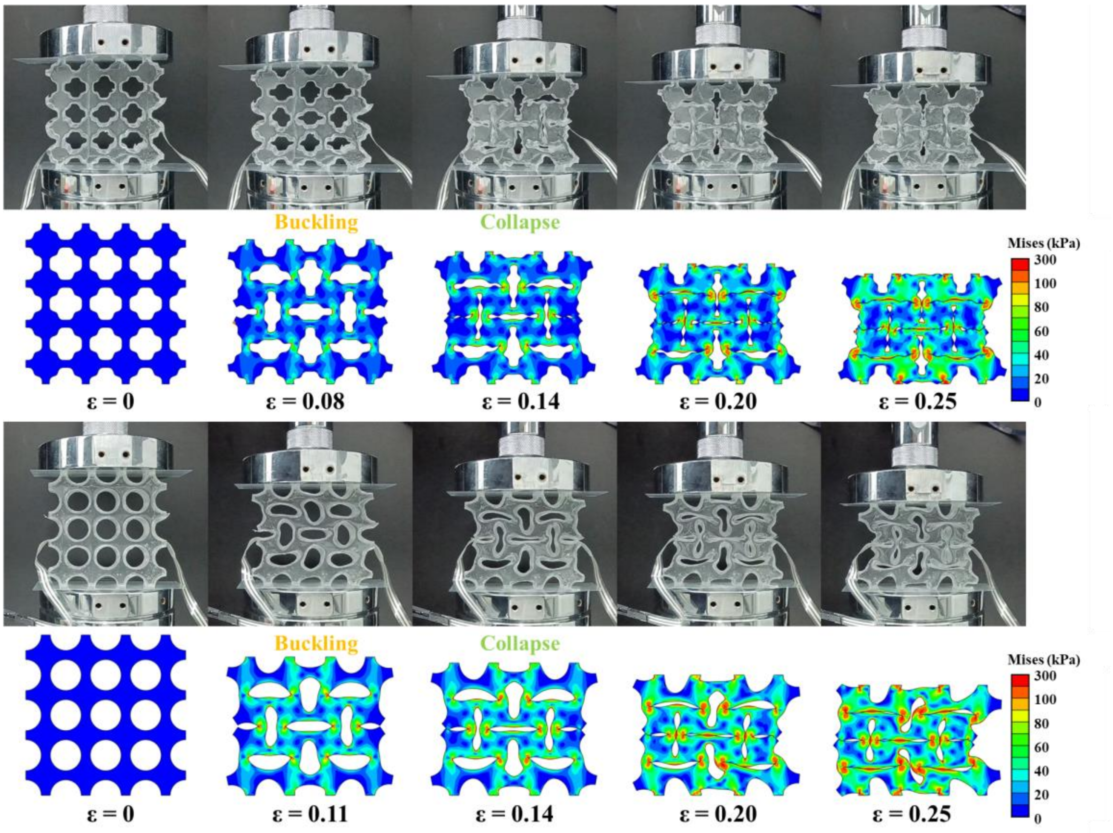

Figure S2. The experimental results and simulated stress distribution at ε = 0, 0.08, 0.14, 0.20 and 0.25 for the MM-F specimen and at ε = 0, 0.11, 0.14, 0.20 and 0.25 for the MM-C specimen.

Supplementary Note 2: Comparison of electro-mechanical characterization of MM-F and MM-C. The two metamaterial specimens are cyclically loaded and unloaded five times with a maximum displacement of 20 mm at the rate of 500 mm/min. The two metamaterial piezoelectric prototypes exhibit a similar voltage-time relationship: a negative voltage peak (V_{min}) occurs at the loading stage and a positive voltage peak (V_{max}) appears at the unloading stage (Figure S3a). The negative voltage peak is the result of a sharp change in strain at buckling. The buckling strains of the two specimens are different, thus, the MM-F and MM-C piezoelectric prototypes generate V_{min} asynchronously. The positive voltage peak is the result of the metamaterial rebound, i.e., the inverse process of the buckling with a slightly lower speed. Consequently, V_{max} is slightly smaller than the absolute value of V_{min}. The average values of the reaction forces corresponding to the minimum and maximal voltages of the last four cycles are used to compare the electro-mechanical performance of the MM-F and MM-C piezoelectric prototypes. When the two piezoelectric films generate V_{min}, the reaction force of the MM-C is significantly larger than that of the MM-F ($P<0.001$), as shown in Figure S3b. The MM-F with PVDF1 generates the same level of voltage as the MM-C with PVDF1 ($P>0.001$) while under a smaller force, and exhibits high output consistency with PVDF1 and PVDF2 (Figure S3c).

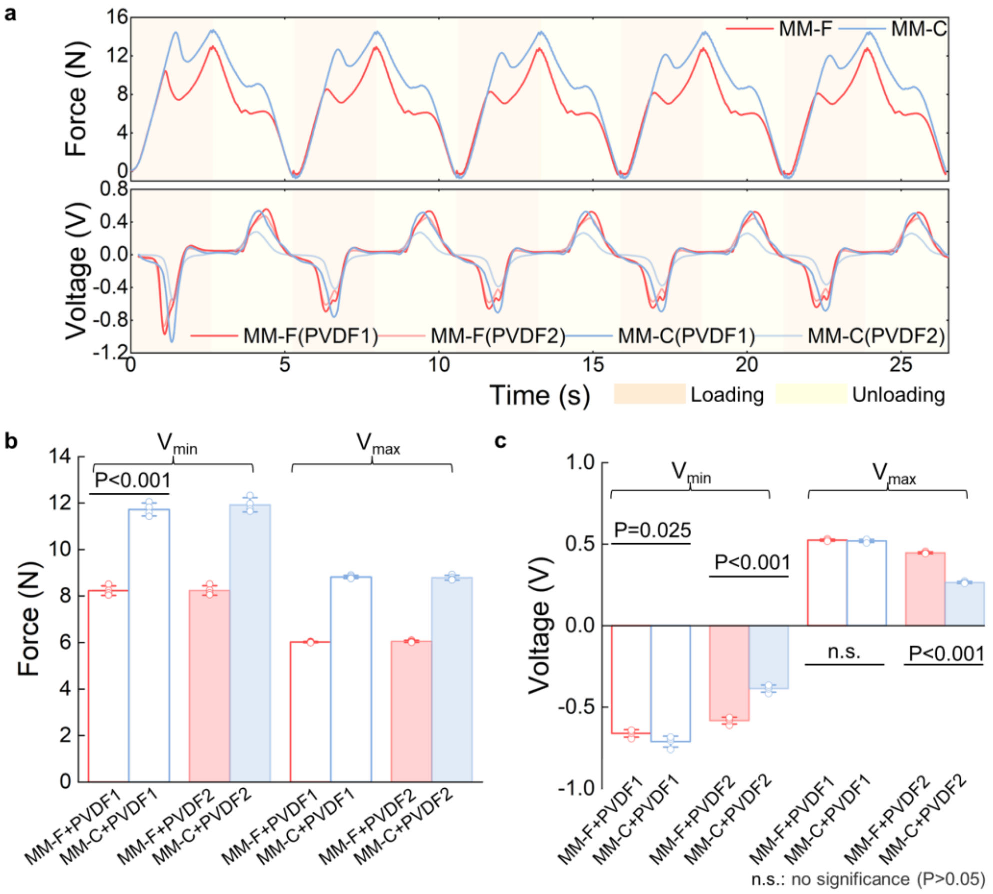

Figure S3. **a** Experimental reaction force and open-circuit voltage of the MM-F and MM-C with the PVDF undergoing five cycles of loading and unloading. **b** The forces endured by the two metamaterials at **c** the minimum and maximum voltages output by the PVDF films. The hollow histograms refer to the data related to PVDF1 and the solid histograms represent the data related to PVDF2. Mean values are shown and error bars represent ±s.d. (n=4 samples per group), as analyzed by one-way ANOVA with post hoc *t*-tests with Bonferroni correction.

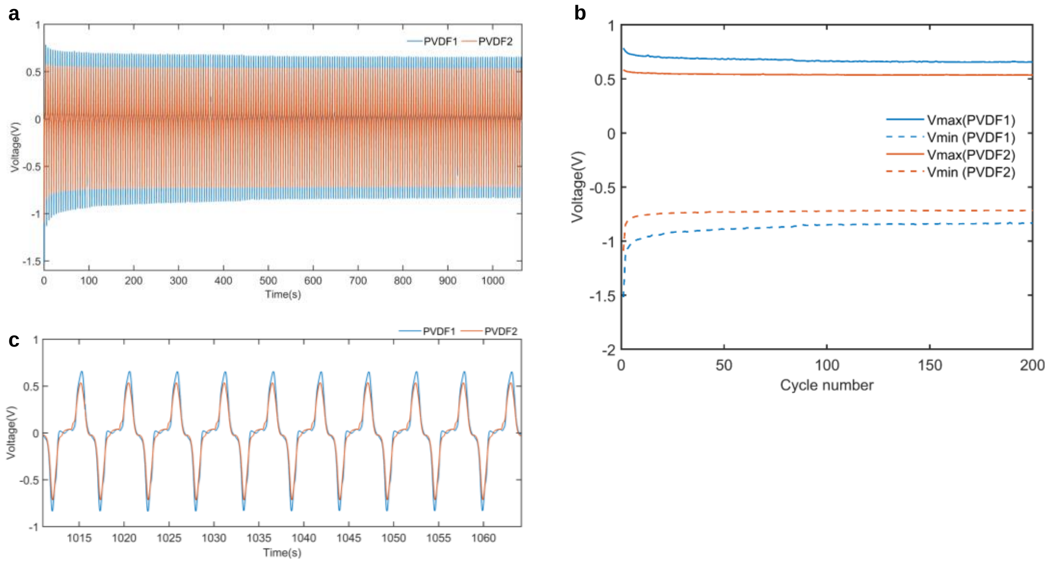

Figure S4. Voltage curves of PVDF1 and PVDF2 with time under 200 cycles of compression. **b** The maximum and minimum voltages of PVDF1 and PVDF2 with time under 200 cycles of compression. **c** Voltage curves of PVDF1 and PVDF2 over time for the last ten cycles of 200 compressions.

Supplementary Note 3: The stability of MM-F specimen under compression deformation of 20 mm at 500 mm/min. After 200 cycles of compression, the maximum and minimum voltages of the MM-F sample are almost constant, as shown in Figure S3a and b. Ignoring the preloading process (the first 20 cycles), the maximum voltages of PVDF1 and PVDF2 decrease by 5.14% and 5.60%, and the minimum voltages decrease by 10.55% and 3.69%, respectively.

Supplementary Note 4: Deformation parameters and velocity parameters of 63 groups of experiments with cyclic compression for 5 times. For deformation of 5, 6, 7, 8 and 25 mm, we conduct tests with six kinds of compression velocity made up of 50, 100, 200, 300, 400 and 500 mm/min. For deformation of 10 mm, we conduct tests with five kinds of compression velocity made up of 50, 100, 200, 300 and 500 mm/min. For deformation of 9, 15 and 20 mm, we conduct tests with nine kinds of compression velocity made up of 50, 100, 200, 250, 300, 350, 400, 450 and 500 mm/min. Note that for 15 mm (400 mm/min)), we conduct two tests.

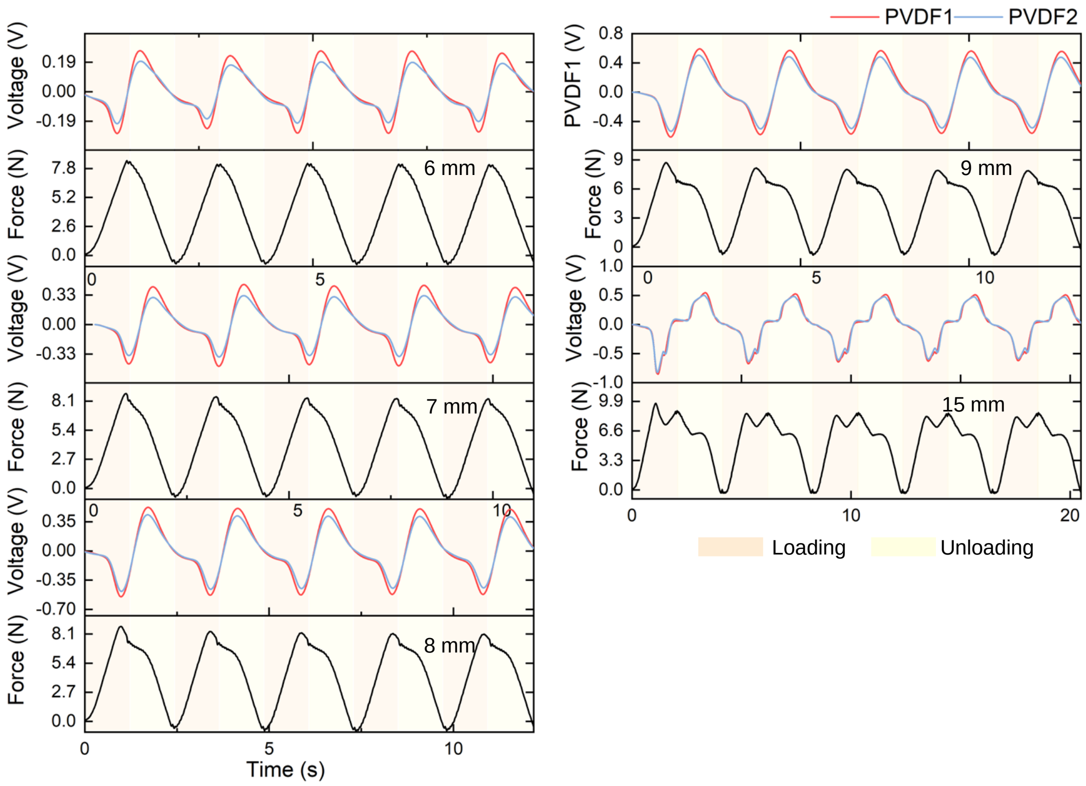

Figure S5. Mechanical and electrical responses for experiments under other 5 different compression amplitudes when compression speed is equal to 500 mm/min.

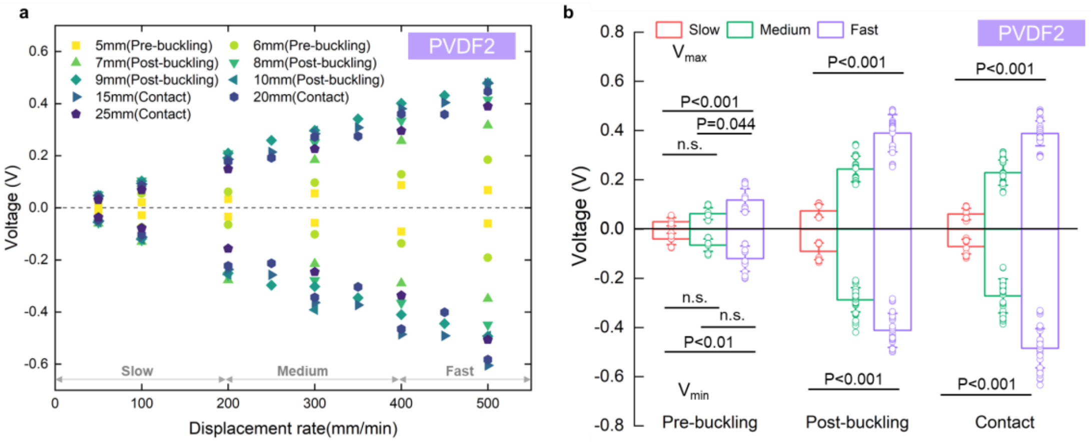

Figure S6. a The values of the maximum and minimum voltages for PVDF2 under different compression displacements and compression velocities. **b** The values of the maximum and minimum voltages for PVDF2 under three types of compression displacements and three types of compression velocities. Mean values are shown and error bars represent ±s.d.(n=16-40 samples per group), as analyzed by one-way ANOVA with post hoc *t*-tests with Bonferroni correction.

Supplementary Note 5: The self-powered mechanism of the mechanical metamaterial piezoelectric device.

The mechanical metamaterial piezoelectric device is composed of a metamaterial and piezoelectric material (PVDF) integrated on both sides of the metamaterial. As an intelligent material, piezoelectric materials possess crystalline structures with positively and negatively charged particles (ions or ion clusters). Under external stress, changes in the molecular structure lead to a nonzero vector sum of internal electric dipoles, resulting in the generation of opposite charges on its surfaces (known as the positive piezoelectric effect). Therefore, by adhering PVDF films to the ligament, the central region of the metamaterial where the maximum strain occurs, the changes in external grip force induce variations in stress within the ligament, consequently leading to changes in surface charge on the piezoelectric material. The variation in surface charge of the piezoelectric material over a grip force measurement cycle is illustrated in **Figure S7**. At the outset, when no grip force is applied to the metamaterial (State 1), there are no changes in surface stress, resulting in no distribution of charge on the piezoelectric material adhered to the ligament. As the grip force gradually increases (State 2), the metamaterial experiences compression due to the pressure exerted on the ligament, leading to a progressive accumulation of surface charges on the compressed piezoelectric material. This continues until the metamaterial's ligament is fully concaved (State 3). During this stage, due to changes in the distribution of surface charges, a potential difference arises, resulting in a current flow from bottom to top. Subsequently, as the grip force diminishes gradually (State 4), the ligament rebounds, returning to its original state. The surface charge on the piezoelectric material progressively diminishes until it reaches its initial state. During this phase, as the potential difference gradually decreases, the direction of current flow reverses, flowing from top to bottom. By harnessing the inherent electromechanical conversion properties of the piezoelectric material and leveraging the influence of grip force on the internal stress distribution within the metamaterial, variations in induced surface charges on the piezoelectric material are achieved. This design enables the grip force device to output electric signals that change in response to grip force variations, all without requiring an external power source. The output electric signals are subjected to machine learning methods for classification and prediction, thereby enabling self-powered grip strength properties sensing.

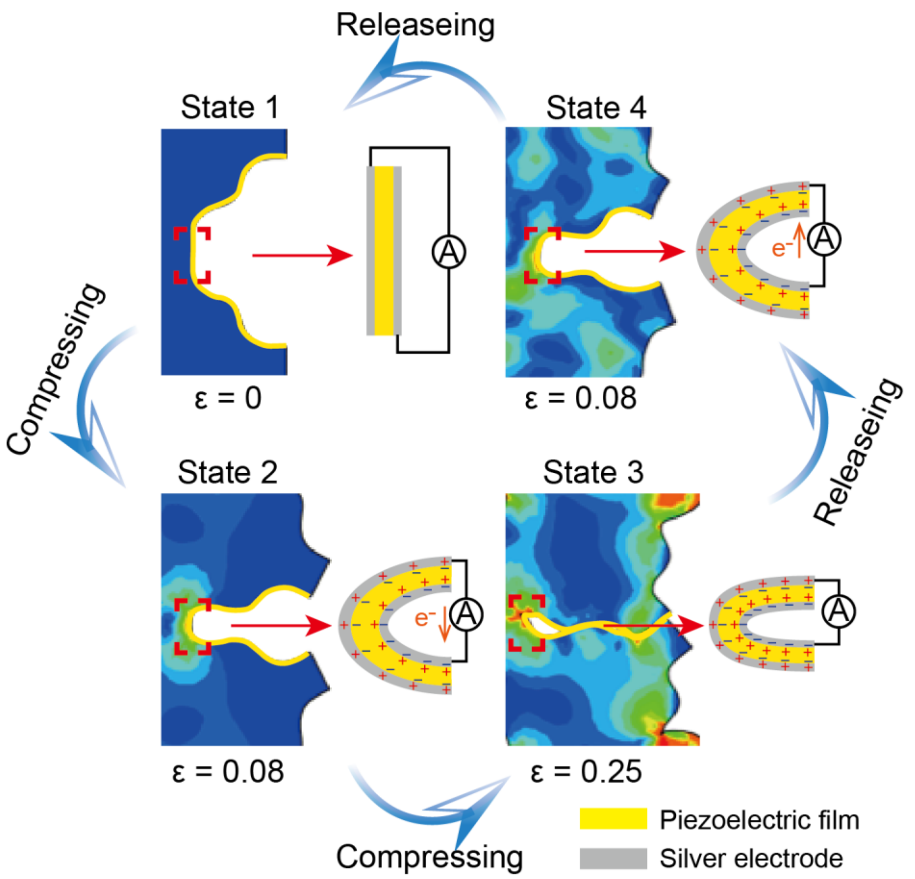

Figure S7 Schematic diagram of PVDF-induced charge variation during a grip measurement cycle.

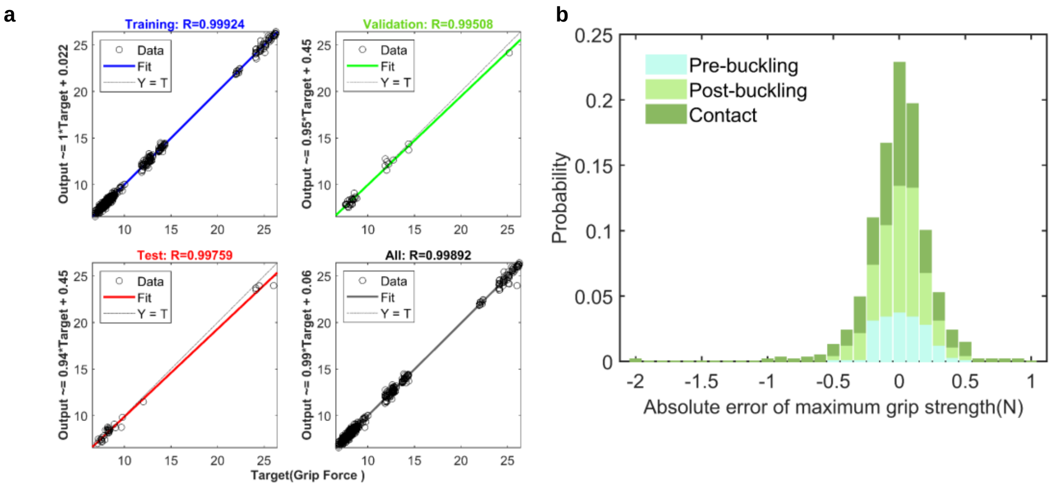

Figure S8. a The linear correlation coefficient between target grip force and output target force. 90%, 5% and 5% of the data sets are used for training, validating and testing, respectively. **b** The absolute error between target grip force and output target force for three types of compression.

Supplementary Note 6: The dimensionality reduction and reconstruction algorithms. Principal component analysis (PCA), aiming at obtaining a hyperplane to express sample points in orthogonal attribute space, is the most commonly used method for dimensionality reduction. We first centralize all samples $\boldsymbol{X}$, then calculate the covariance matrix of the samples and perform the eigenvalue decomposition. Then the eigenvectors corresponding to the largest d_1 eigenvalues form the projection matrix $\boldsymbol{W}$. We set a reconstruction threshold t from the perspective of reconstruction, also known as information cumulative contribution rate, and then select the minimum d_1 value that makes the following formula valid:

$$\frac{\sum_{i=1}^{d_1} \lambda_i}{\sum_{i=1}^{d} \lambda_i} \geq t$$

By retaining projection matrix $\boldsymbol{W}$ and sample mean vector $\overline{\boldsymbol{X}}$, samples can be projected to low-dimensional space through simple vector subtraction and matrix-vector multiplication. Meanwhile, the samples in low-dimensional space can also be reconstructed in high-dimensional space by discarding part of the information, as shown in Figure S8.

Coefficient of determination R^2 is used to judge the fitting results between the reconstructed force curve and the original force curve:

$$R^2 = 1 - \frac{\sum_{i=1}^{n}(y_i - Y_i)^2}{\sum_{i=1}^{n}(y_I - \bar{Y})^2}$$

where n is the total number of points on the curve, y_i is the fitting value of the sequence Y_i and $\bar{Y}$ is the mean of the sequence Y_i. The closer R^2 is to 1, the better the fitting is.

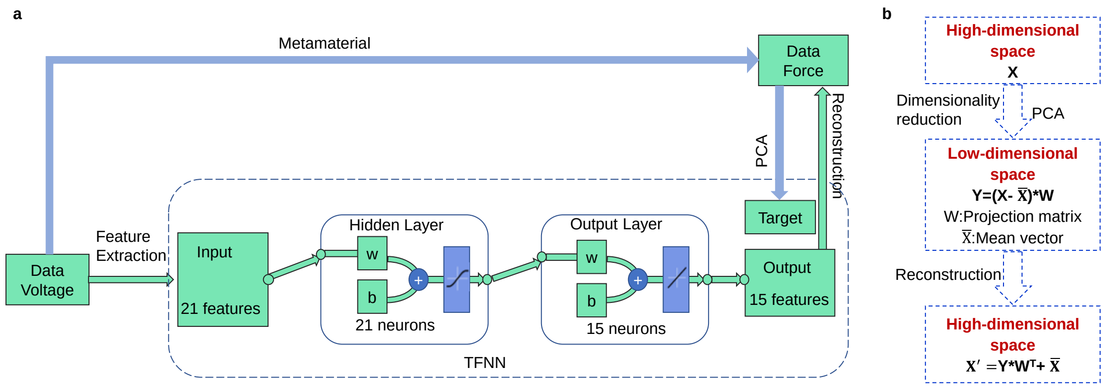

Figure S9. a The flow chat of machine learning used to map from voltage data to 15 main features of force data. **b** The flowchart about dimensionality reduction and reconstitution.

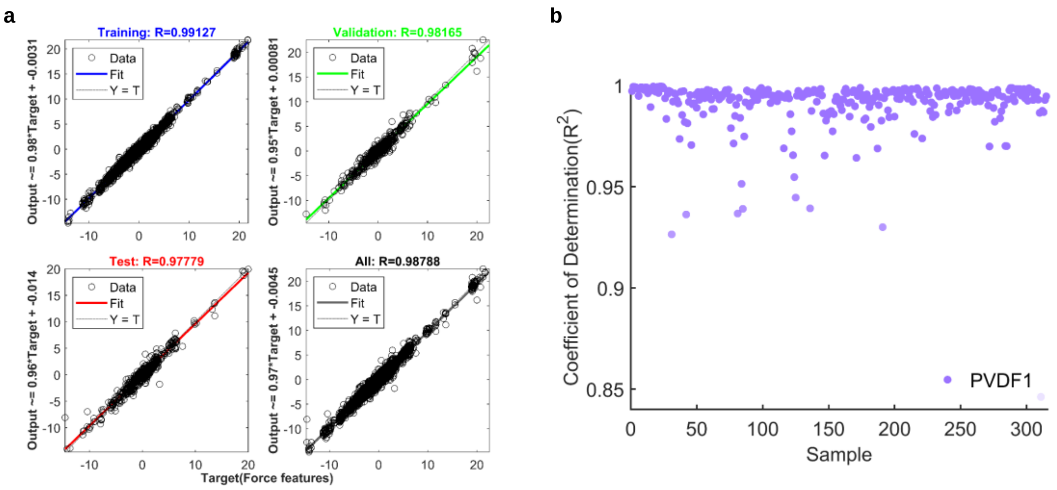

Figure S10. a The linear correlation coefficient of training, validation, test and all datasets between the output (15 main features of force) of the neural network after training and the actual target output. 70%, 15% and 15% of the data sets are used for training, validating and testing, respectively. **b** Coefficient of determination R^2 between the original force curve and the reconstructed force curve for all samples of PVDF1.

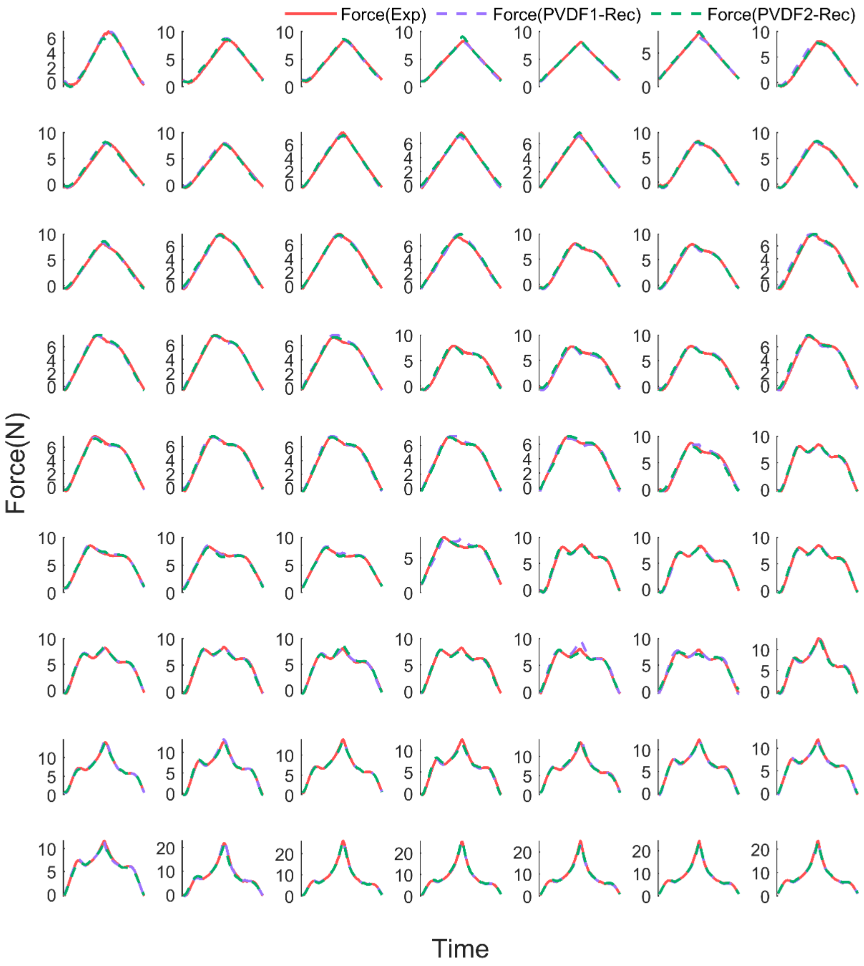

Figure S11. The reconstructed forces of PVDF1 and PVDF2 compared to experimental force for 63 groups of experiments. From left to right and top to bottom, compression displacements and corresponding speeds are 5 mm (500, 400, 300, 200, 100 and 50 mm/min), 6 (500, 400, 300, 200, 100 and 50 mm/min), 7 mm (500, 400, 300, 200, 100 and 50 mm/min), 8 mm(500, 400, 300, 200, 100 and 50 mm/min), 9 mm (500, 450, 400, 350, 300, 250, 200, 100 and 50 mm/min), 10 mm (500 mm/min), 15 mm (400 mm/min), 10 mm (300, 200, 100 and 50 mm/min) , 15 mm (500, 450, 400, 350, 300, 250, 200, 100 and 50 mm/min), 20 mm (500, 450, 400, 350, 300, 250, 200, 100 and 50 mm/min) and 25 mm (500, 400, 300, 200, 100 and 50 mm/min).

Supplementary Note 7: As shown in Figure S17, in the demonstration experiment, the buttons "MM-F (30:1)", "MM-F (10:1)", "Test" and "Save" are used to select different metamaterials for grip strength detection and store relevant data. The figure window "Contact state" determines the metamaterial deformation state (Pre-buckling, Post-buckling or Contact) under the grip action according to the current measured voltage signal and displays a representative picture of the deformation state. The axes graphics objects "Voltage Curve" and "Grip Curve" display the current measured voltage signal and the grip force curve reconstructed by the machine learning algorithm. The dynamic text box "Grip Velocity" and "Grip Strength" infer the grip speed under the grip action and the value of the grip strength according to the machine learning algorithm.

Supplementary Table 1: Basic information of the participants (*n* = 9)

(Dominant hand)	Gender	Age	Height(cm)	Weight(kg)	BMI(kg/m^2)	Health condition
Common Elderly						
1(R)	Male	58	165	70	25.71	
2(L)	Male	55	168	55	19.49	Cholecystectomy
3(R)	Female	60	156	70	28.76	
4(L)	Female	53	160	65	25.39	
5(R)	Female	55	158	60	24.03	Hypertension
6(R)	Male	67	167	62	22.23	Hypertension
7(L)	Female	60	168	58	20.55	
Elderly patients with schizophrenia						
1(R)	Female	62	159	53	20.96	Schizophrenia
2(R)	Female	63	167	68	24.38	Schizophrenia

BMI = Body mass index (BMI).

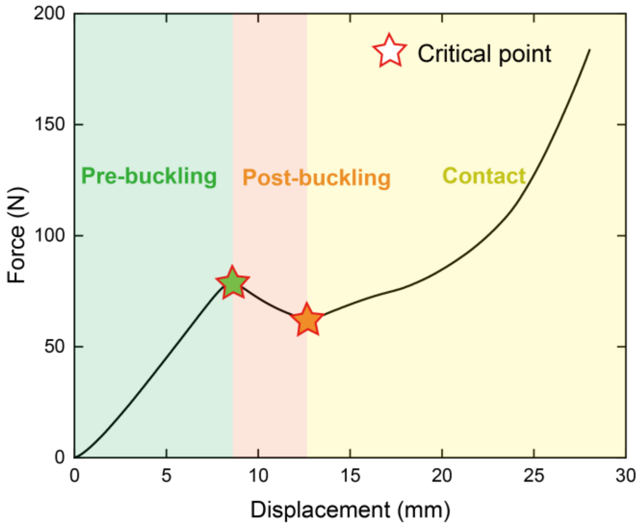

Figure S12 The experimental force-displacement curve of the MM-F (PDMS 10:1) specimen. The buckling and collapse points are located in 8.6 mm and 12.7 mm.

Supplementary Note 8: Deformation parameters and velocity parameters of 14 groups of experiments for MM-F (PDMS 10:1) with cyclic compression for 5 times. For deformation of 5, 6, 7, 8, 9, 10, 15, 20 and 25 mm, we conduct tests under compression velocity of 100, 200, 300, 400 and 500 mm/min. The machine learning performance is shown in Figure S13 and 14. The composition ratio of PDMS has negligible influence on the performance of the machine learning model. As illustrated in Figure S14, the recognition accuracies for the three types of deformation and the three speed levels are 100% and 97.3%, respectively(Figure 13a, b). The relative error between the output maximum force and the actual target maximum force is 0.21 ± 2.43% (mean ± std) and ranges between -3.42% and 4.04% (95th percentiles) (Figure 13c). 95.33% of the coefficients of determination R^2 between the original force curve and the reconstructed force curve using the voltages of PVDF1 or PVDF2 are greater than 0.95 (Figure 14d), indicating an excellent reconstruction performance.

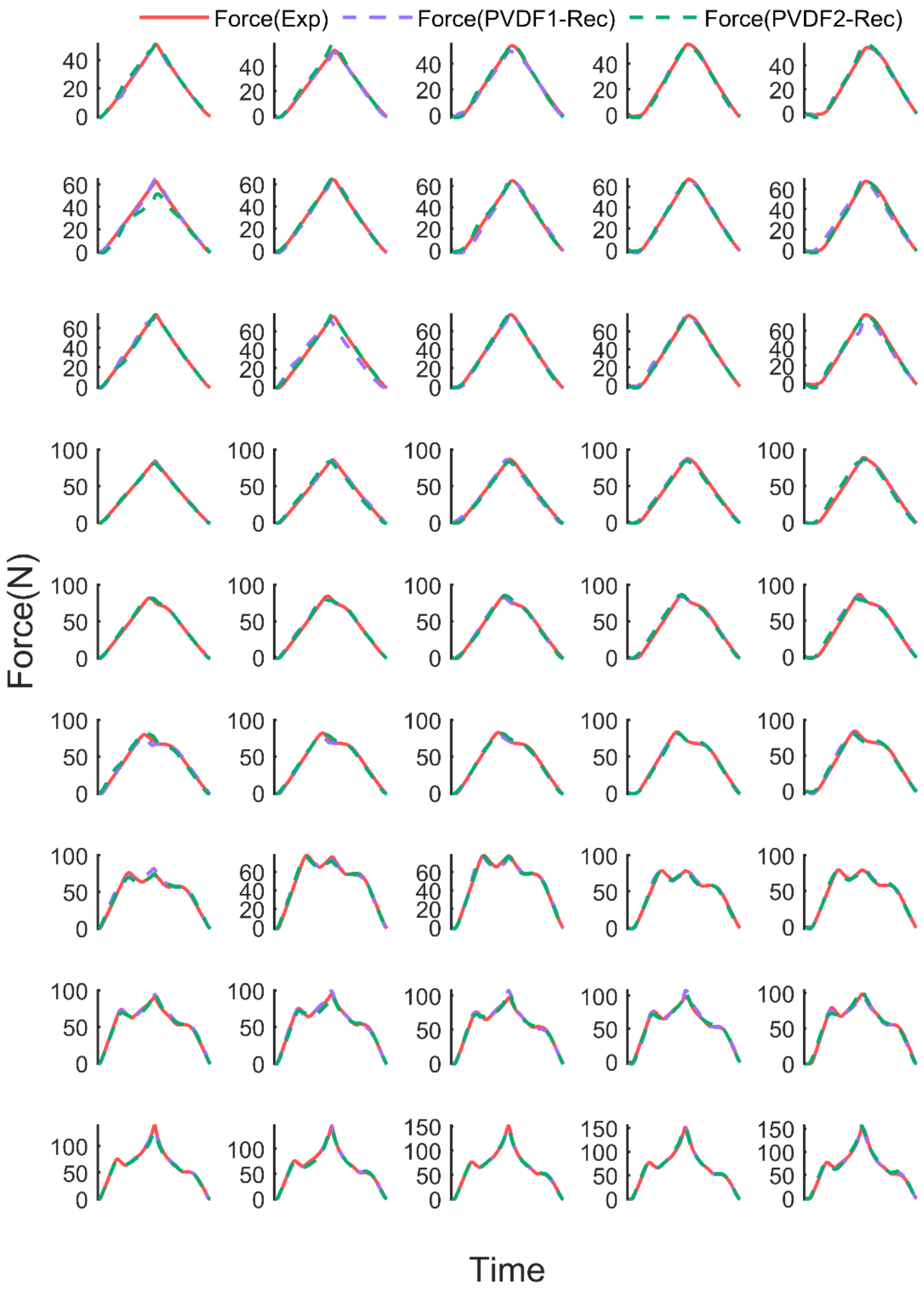

Figure S13. The reconstructed forces for MM-F (PDMS 10:1) of PVDF1 and PVDF2 compared to experimental force for 45 groups of experiments. From left to right and top to bottom, compression displacements and relative speeds are 5 mm (100, 200, 300, 400 and 500 mm/min), 6 mm (100, 200, 300, 400 and 500 mm/min), 7 mm (100, 200, 300, 400 and 500 mm/min), 8 mm (100, 200, 300, 400 and 500 mm/min), 9 mm (100, 200, 300, 400 and 500 mm/min), 10 mm (100, 200, 300, 400 and 500 mm/min), 15 mm (100, 200, 300, 400 and 500 mm/min), 20 mm (100, 200, 300, 400 and 500 mm/min) and 25 mm (100, 200, 300, 400 and 500 mm/min).

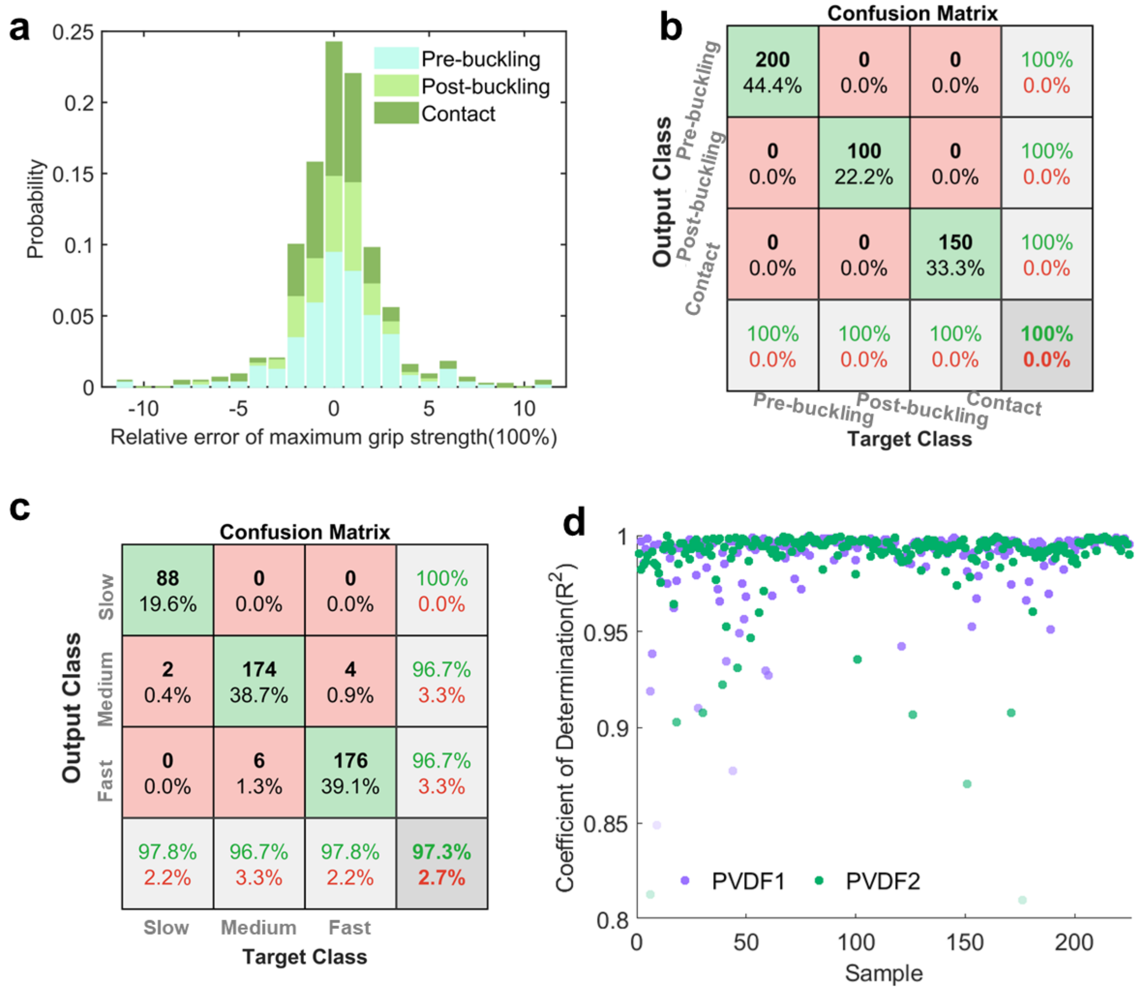

Figure S14. Training results for MM-F (PDMS 10:1). a Probability distribution of relative errors in maximum grip strength measurements. **b** The confusion matrix for recognition of three types of electromechanical classifications. **c** The confusion matrix for recognition of three types of compression speed. The recognition accuracies of training set (90%) and test set (10%) are both 100%. **d** Coefficient of determination R^2 between the original force curve and the reconstructed force curve for all samples. The recognition accuracies of training set (90%) and test set (10%) are 98.0% and 91.1%, respectively.

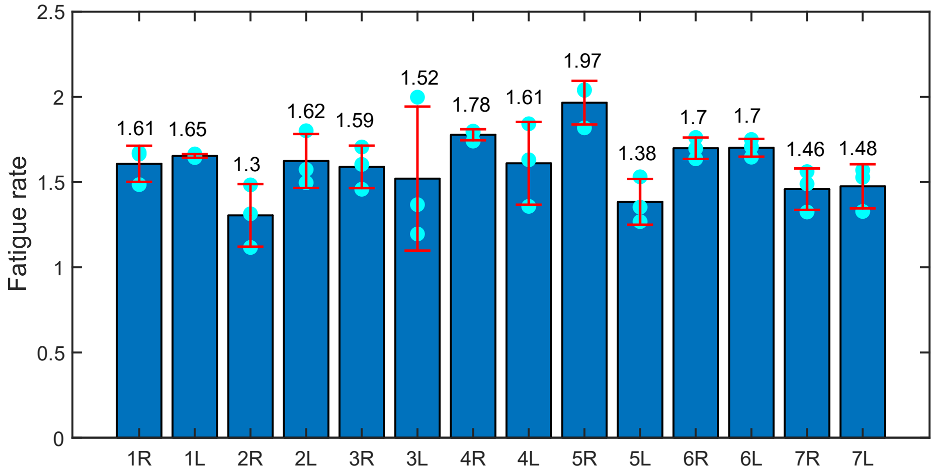

Figure S15. The fatigue rate derived from the grip strength curve.

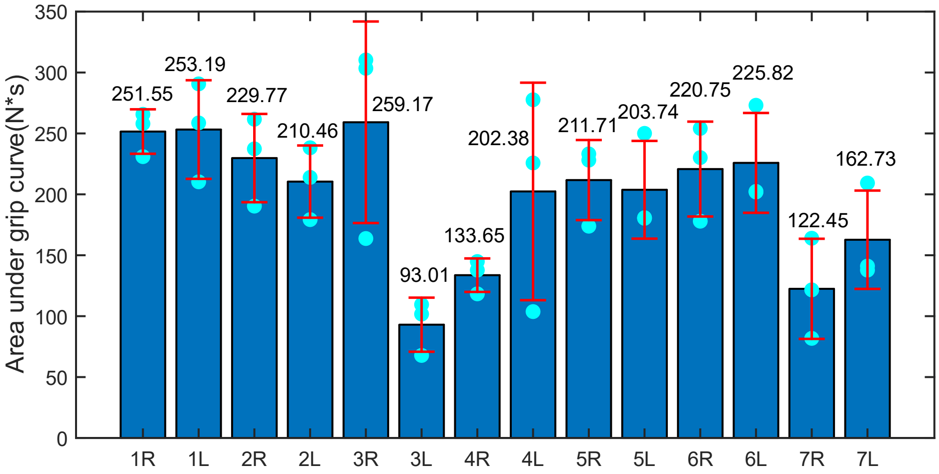

Figure S16. The area under curve derived from the grip strength curve.

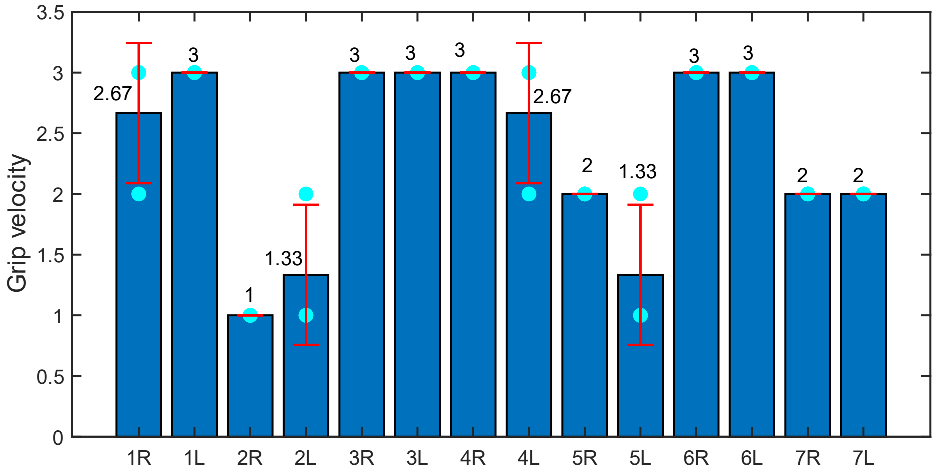

Figure S17. The grip velocity derived from the grip strength curve. 1 represents Slow, 2 represents Medium and 3 represents Fast.

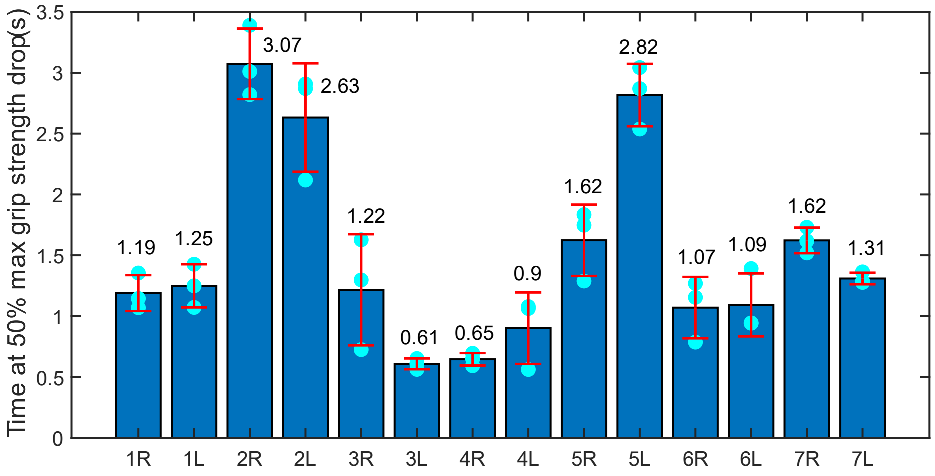

Figure S18 The amount of time it takes for grip strength to drop to 50% of maximum grip strength.

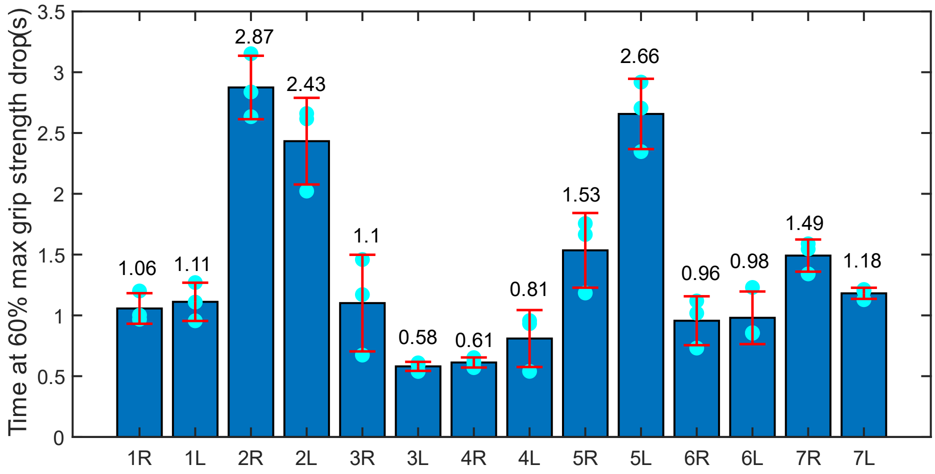

Figure S19 The amount of time it takes for grip strength to drop to 60% of maximum grip strength.

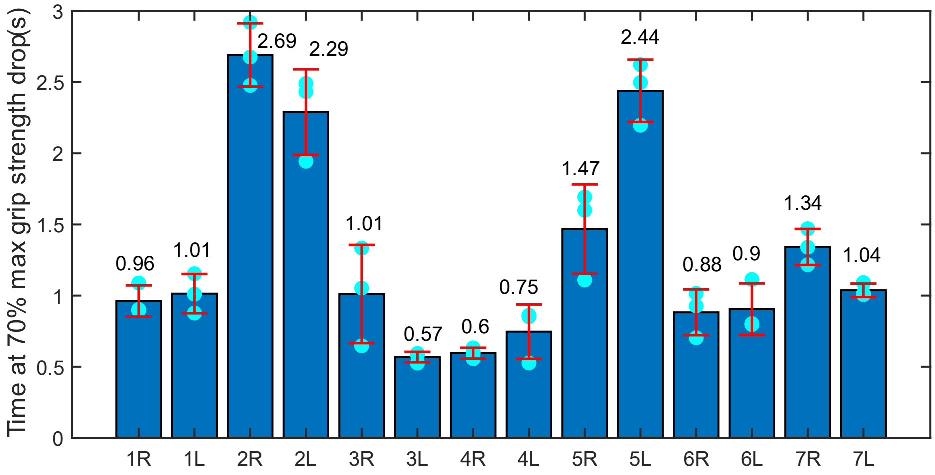

Figure S20 The amount of time it takes for grip strength to drop to 70% of maximum grip strength.

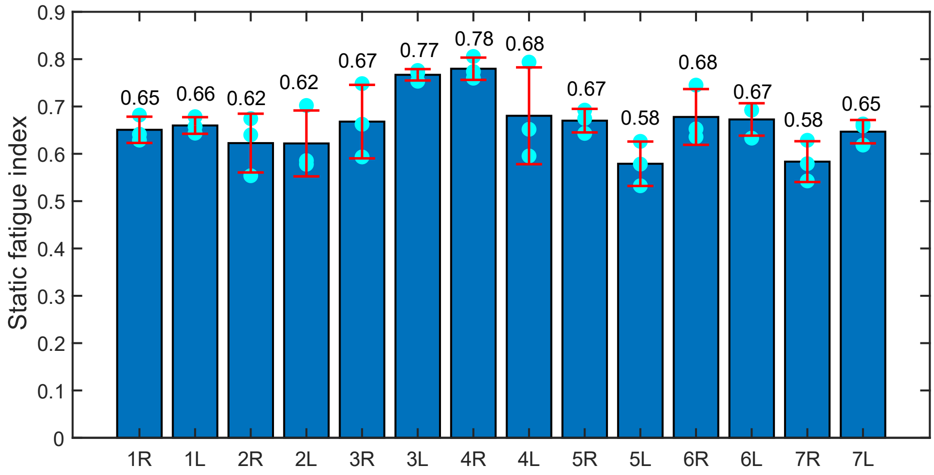

Figure S21 Static fatigue rate derived from the grip strength curve. Static fatigue rate=1-AUC/HAUC. AUC is the integral of grip strength over the interval [T_{max}, T_{total}]. HAUC is equal to maximum grip strength times T_{total}-T_{max}. T_{max} is defined as the time when maximum grip strength occurs. T_{total} is defined as the total time of a single grasp.

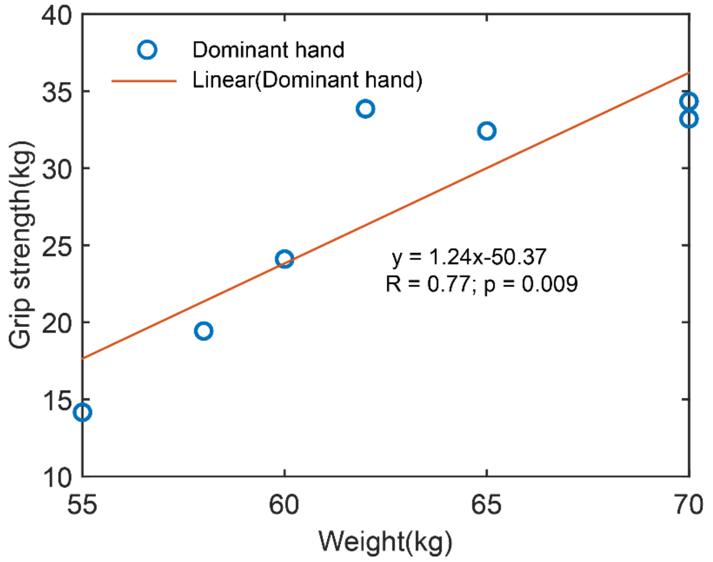

Figure S22. Pearson correlation (Weight vs. Dominant hand).

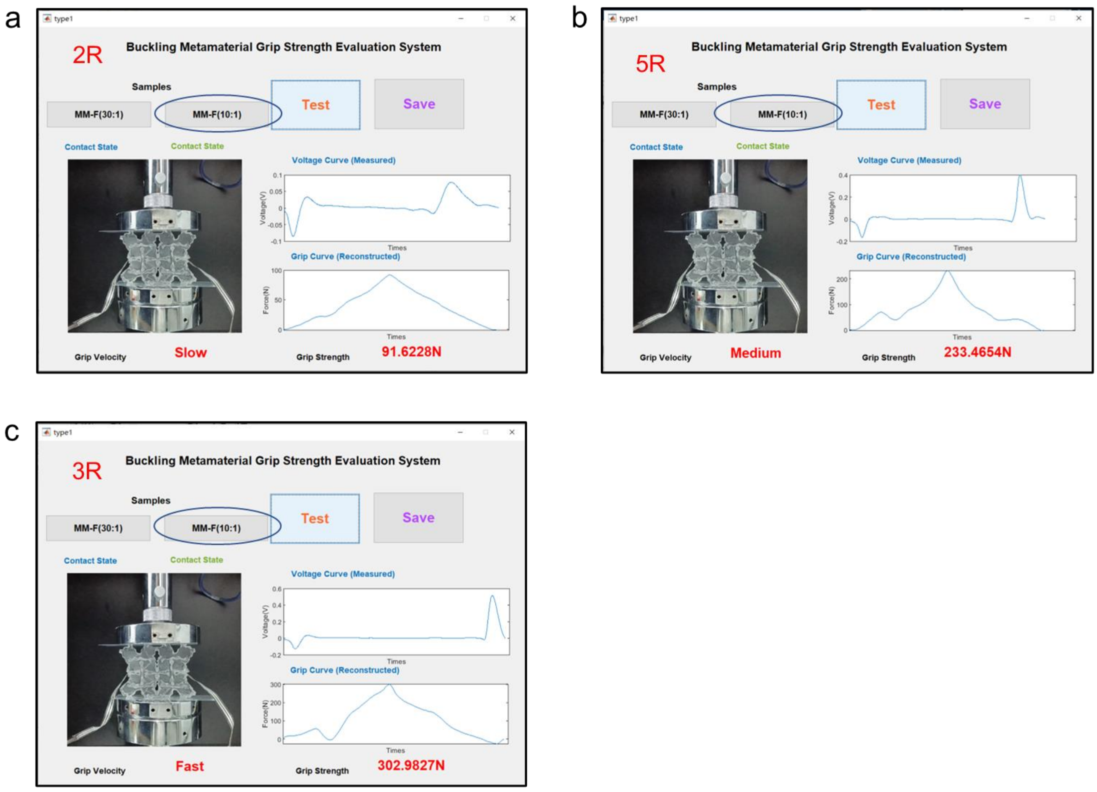

Figure S23. The GUIs for participants 2,5 and 3 testing by MM-F (10:1). All test the right hand.

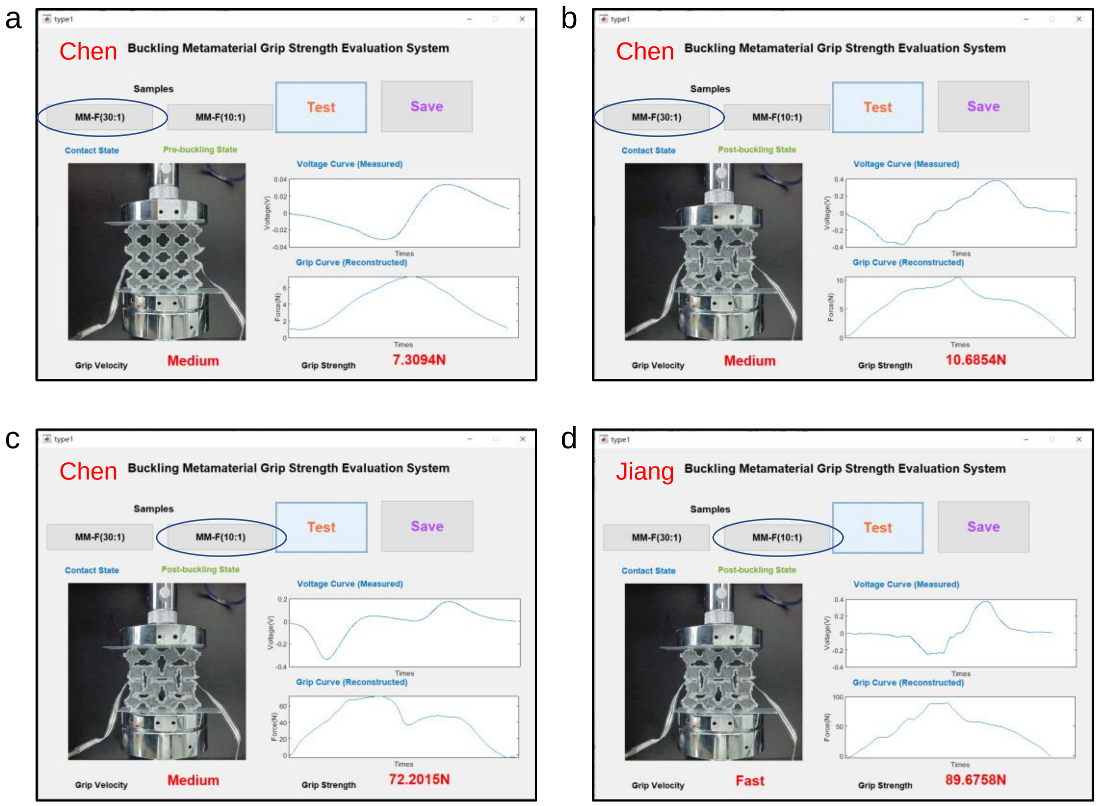

Figure S24. a The test results under pre-buckling state with medium velocity for MM-F (30:1). **b** The test results under post-buckling state with medium velocity for MM-F (30:1). **c** The test results under post-buckling state with medium velocity for MM-F (10:1). **d** The test results under post-buckling state with fast velocity for MM-F (10:1).

Supplementary Table 2: Comparison of experimental conditions between actual test and static machine test

Experimental condition	Static machine test (Calibration data)	Actual test (Measurement data)
Direction of motion	Unidirectional	Unidirectional
Motion constraint	Linear guide	Linear guide
Contact condition	Surface contact	Surface contact
Contact surface material	Metal (Rigid)	Resin (Rigid)

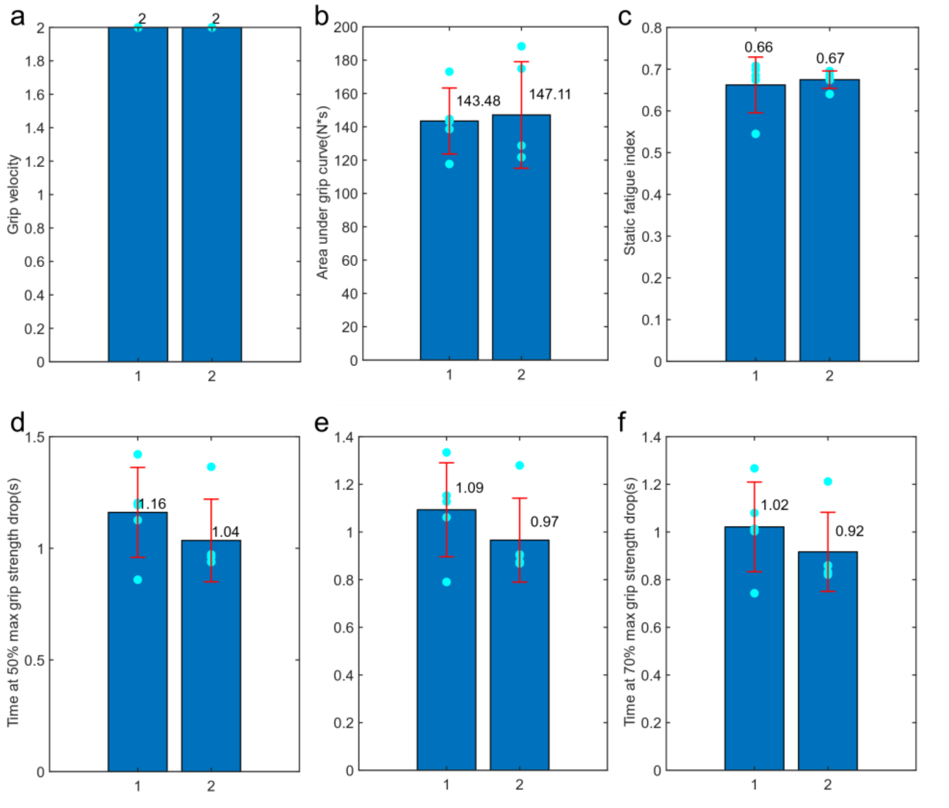

Figure S25 The grip velocity (a), the area under the curve (b), the static fatigue rate (c), the amount of time it takes for grip strength to drop to 50% (d),60% (e) and 70% (f) of maximum grip strength for two female schizophrenic patients.

References

[1] Overvelde, J. T. B., Shan, S. & Bertoldi, K. Compaction through buckling in 2D periodic, soft and porous structures: effect of pore shape. *Adv. Mater.* **24**, 2337–2342 (2012).

[2] Overvelde, J. T. B. & Bertoldi, K. Relating pore shape to the non-linear response of periodic elastomeric structures. *J. Mech. Phys. Solids* **64**, 351–366 (2014).

Supplementary Videos

Movie S1: Nominal strain-stress curves of MM-F and MM-C.

Movie S2: Real-time graphical user interface for medical diagnosis.

Movie S3: Interacted training games for rehabilitation therapy.
